# An automated decision-making procedure for ranking and selecting species in biodiversity projects

**DOI:** 10.1038/s44185-026-00140-x

**Published:** 2026-05-30

**Authors:** Torsten H. Struck, Thomas Marcussen, Astrid Böhne, Rosa Fernández, José Melo-Ferreira, Isabelle Florent, Carmela Gissi, Christian de Guttry, Jennifer A. Leonard, Seanna McTaggart, Camila J. Mazzoni, Rita Monteiro, Olga Vinnere Pettersson, João Pimenta, Jaakko Pohjoismäki, Katja Reichel, Andrii Tarieiev, Rebekah A. Oomen

**Affiliations:** 1 Natural History Museum, University of Oslo, Oslo, Norway; 2 Centre for Ecological & Evolutionary Synthesis, University of Oslo, Oslo, Norway; 3 Leibniz Institute for the Analysis of Biodiversity Change, Museum Koenig Bonn, Bonn, Germany; 4 Metazoa Phylogenomics and Genome Evolution Lab, Institute of Evolutionary Biology (CSIC-UPF), Barcelona, Spain; 5 CIBIO, Centro de Investigação em Biodiversidade e Recursos Genéticos, InBIO Laboratório Associado, Universidade do Porto, Vairão, Portugal; 6 Departamento de Biologia, Faculdade de Ciências, Universidade do Porto, Porto, Portugal; 7 BIOPOLIS Program in Genomics, Biodiversity and Land Planning, CIBIO, Vairão, Portugal; 8 UMR7245, National Museum of Natural History and CNRS, Paris, France; 9 Dipartimento di Bioscienze, Biotecnologie e Ambiente, Università degli Studi di Bari “Aldo Moro”, Bari, Italy; 10 Institute of Biomembranes, Bioenergetics and Molecular Biotechnologies, CNR, Bari, Italy; 11 CoNISMa, Consorzio Nazionale Interuniversitario per le Scienze del Mare, Roma, Italy; 12 SIB Swiss Institute of Bioinformatics, Environmental Bioinformatics group, Amphipôle, Quartier UNIL-Sorge, 1015 Lausanne, Switzerland; 13 Conservation and Evolutionary Genetics Group, Estación Biológica de Doñana (EBD-CSIC), Seville, Spain; 14 Earlham Institute, Norwich Research Park, Norwich, United Kingdom; 15 Department of Evolutionary Genetics, Leibniz Institute for Zoo- and Wildlife Research (IZW), Berlin, Germany; 16 Berlin Center for Genomics in Biodiversity Research (BeGenDiv), Berlin, Germany; 17 Science for Life Laboratory - Sweden (SciLifeLab), National Genomics Infrastructure, Uppsala University, Uppsala, Sweden; 18 Department of Environmental and Biological Sciences, University of Eastern Finland, Joensuu, Finland; 19 Dahlem Center of Plant Sciences, Institute of Biology, Freie, Universität Berlin, Berlin, Germany; 20 Institute of Biology, Geobotany and Botanical Garden,, Martin Luther University Halle-Wittenberg, Halle (Saale), Germany; 21 Department of Forest Genetics and Forest Tree Breeding, University of Göttingen, Göttingen, Germany; 22 Department of Biological Sciences, University of New Brunswick, Saint John, Canada; 23 Tjärnö Marine Laboratory, University of Gothenburg, Gothenburg, Sweden; 24 Centre for Coastal Research, University of Agder, Kristiansand, Norway

**Keywords:** Computational biology and bioinformatics, Ecology, Ecology, Genetics

## Abstract

In large-scale biodiversity genomics projects, the number of species that could be sequenced exceeds the resources available. Species selection is therefore a crucial component, requiring clear criteria and procedures. In a bottom-up approach, the Biodiversity Genomics Europe (BGE) project implemented an Automated Decision-Making (ADM) process for species selection based on objective criteria and tested it on simulated and empirical data. Here, we present this species ranking ADM process, which includes three stages: exclusion, ranking, and feasibility check. The composition of selected species retained the diversity of the community-nominated species pool for key taxonomic, geographic, and demographic assessment criteria while reducing bias. Feasibility and funding limits influenced the final selection more than other factors, indicating that investments in these areas would improve available reference-genome diversity. The ADM achieved species selection for genome sequencing in a large-scale biodiversity project in a relatively objective manner consistent with the broader European biodiversity genomic community’s priorities.

## Introduction

Facing the human-driven sixth mass extinction^1^, there is an urgent need for mapping biodiversity on the genomic level^2,3^. Such data would increase our understanding of evolutionary relationships and ecological interactions, and enable more effective conservation and restoration efforts^2^. With the Earth Biogenome Project (EBP), Lewin et al. ^2^ suggested a moonshot for biology: sequencing a reference genome for each eukaryotic species on Earth within the next 10 years to support biodiversity genomic research at all levels. The EBP and associated large-scale genome sequencing initiatives^2–9^ aim at accomplishing this goal. These large-scale biodiversity projects often have a regional or taxonomic scope. For example, the Vertebrate Genomes Project (VGP) aims at sequencing only vertebrate species^4^ partially funded by different sources, while the Darwin Tree of Life (DToL) project will sequence only eukaryotic species of the bioregion of Britain and Ireland^5^ funded by the Wellcome Trust and the Earth BioGenome Project Norway (EBP-Nor) only Norwegian species^7^ funded by the Research Council of Norway in the first phase. Another genome initiative is the European Reference Genome Atlas (ERGA), which has been established as a bottom-up grassroots movement targeting all eukaryotic biodiversity of Europe while heavily investing in community support^8^. Mostly from within ERGA, the EU project Biodiversity Genomics Europe (BGE) was developed. One of its goals was to establish the distributed generation of reference genomes for species occurring in Europe, i.e., continental Europe, its surrounding islands, and the international waters of the Mediterranean Sea, the Northern Atlantic Ocean east of the Atlantic Ridge, and the Arctic Ocean adjacent to the European continental shelf.

Nonetheless, this is an ambitious goal in all terms, also economically, despite the plummeting costs for genome sequencing. Hence, the plan for accomplishing it has three phases: In the first phase, at least one representative species from each of the about 9,000 families should be targeted. In the second phase, a representative of each of the about 150,000 genera is the target, and then finally each species. Hence, this envisioned a systematics-driven selection process of the species to be targeted. The financial support was to be obtained at the regional and national levels. However, the projects have different degrees of secured funding. Moreover, the number of potential target species is, in general, much higher than the actual number of species that can be processed within finite budgets and limited sequencing capacity. Consequently, this requires species to be prioritized and selected even within large-scale sequencing projects by a decision-making procedure, which can be based on numerous different criteria^10,11^.

Criteria that are potentially relevant for the ranking of candidate species for genome sequencing can be classified into three major categories: biological, technical, and social^10,11^. Biological criteria involve characteristics of the species itself, such as taxonomic representation, genome size, and characteristics of the available specimen(s), such as karyotype (sex), or geographical distribution. Technical criteria, such as those related to sampling procedures like access to liquid nitrogen or maintenance of a cold chain can also be crucial. On the other hand, social criteria involving political or strategic decisions within a consortium, such as regional and institutional representation, can also be relevant in the process. These criteria can also conflict with one another. For example, the goal can be to sample the taxonomic breadth of the eukaryotic tree, but the protocols to generate a reference genome require an amount of tissue not always available. Similarly, projects can prioritize endemic species and prioritize inclusivity and capacity building by involving research teams from countries with lower capacities to sequence reference genomes. However, countries with high numbers of endemic species do not necessarily have lower capacities to sequence genomes, and vice versa. Accordingly, even though objective criteria are applied, subjective components can enter the decision process.

Ideally, species selection procedures should be transparent and as objective as possible despite inherent subjective biases. From other decision procedures, such as acceptance of a scientific publication or hiring for scientific positions, it is known that such biases can influence the decision. For example, there can be intrinsic advantages for English as a first language speakers in scientific activities^12^, as well as gender biases^13^. Similarly, biases can exist towards more iconic and better-known taxa, or economically important taxa, which is to the detriment of conservation goals^14^ and taxonomic representation. Automated decision-making (ADM) processes are widely used across various fields in society to ensure consistency, efficiency, and accuracy in decision-making when the number of criteria is high and the evaluation is complex^15,16^. Such ADM processes can facilitate species selection to be as transparent and objective as possible while acknowledging subjective priorities.

For ADM processes, a distinction may be made between *prioritization* procedures, by which targets are queued for action, and *ranking* procedures, by which targets are ordered for evaluation. Automated prioritization procedures are used in e.g., emergency room triage, cybersecurity threat detection, and task management systems to continuously ensure that high-value or urgent items receive attention first^17^. Automated ranking procedures are used to sort options based on pre-defined criteria such as value or relevance, e.g., in search engines, recommendation systems, and accreditation rankings^18,19^. The purpose of implementing ADM processes is to save time, reduce human unconscious biases and ensure a higher degree of consistency in decision-making. Specifically, the implementation of an ADM requires a set of clearly defined criteria. The selection of this set of criteria can be achieved through mechanisms such as a general vote or other bottom-up, transparent decisions. We therefore argue that an ADM process may ease resource allocation in biodiversity genome projects, while also making the process more transparent and democratic.

The Biodiversity Genomics Europe (BGE) project (EU Horizon Europe BGE Project, Grant Agreement No 101059492, https://biodiversitygenomics.eu/) was based on large community involvement^20^. The aim of BGE was to enable the generation and usage of genomic resources (i.e., DNA barcodes and reference genomes) in biodiversity research. These resources can “enhance understanding of biodiversity, monitor biodiversity change, and guide interventions to address its decline”^20^. Among others, one of the overarching goals was to generate reference genomes from eukaryotic species across Europe to a total genome size of 450 Gigabases (Gb). This was tried to be accomplished in three ways: by supporting case studies in biodiversity research, by sampling from European biodiversity hotspots, and through a community-driven approach to specimen collection. In the latter approach, scientists nominated a species and, if selected by BGE, provided samples/specimens. For this task, depending on the total genome size BGE wanted to sequence reference genomes of up to 150 Gb. To this aim, BGE created a transparent ADM process for selecting species based on a set of a priori objective criteria applied to the pool of nominated species.

To develop the ADM process and its criteria, BGE leveraged its foundation in the European Reference Genome Atlas (ERGA) community and its experience with a community-based pilot project^10,11^. The ERGA community brings together broad taxonomic expertise across the eukaryotic tree of life, along with deep knowledge of the laboratory methods needed for sequencing and assembling reference genomes. Moreover, through its governance structure, which includes a council of national representatives, ERGA stands for a broad representation across Europe and democratic decisions.

Here, we present the ADM process for ranking nominated species from across Europe as implemented by BGE. We describe (1) a community-defined set of ranking criteria, (2) a reproducible ranking algorithm, and (3) a validation of the algorithm by simulated and empirical data. To our knowledge, such an ADM-based ranking process is unique among biodiversity genomics projects. Notably, this ADM process was decided a priori in an open, bottom-up process. Further, its flexible structure and criteria can be tailored to the priorities and feasibility constraints of a given project. Therefore, we anticipate that this procedure developed within BGE has value to other large-scale biodiversity projects, and we provide guidance for other consortia.

## Results

### Automated species selection process

A prerequisite for any selection procedure is that the number of candidates exceeds the number that can be processed. Within the available funding for the community-driven BGE task, sequencing was limited to a total of 150 Gb of estimated haploid genome size. To obtain a list of candidate species for genome sequencing we held two consecutive open calls, in which scientists and citizens could nominate European species for reference-genome sequencing. This nomination was completed by filling in an online questionnaire with information that was later used to rank the species (see “Questionnaire for the nomination in round #2” in the Supplementary Information). The nomination process resulted in a list of 593 species nominations, and, based on the information provided in the nomination forms, summed to an estimated haploid genome size of about 1000 Gigabases (Gb). This meant that the total genome size of the nominations was ca. seven times higher than the project’s capacity. After the ranking procedure, 99 species (17% of the total) with a combined projected 150 Gb genome size were selected for genome sequencing.

**Fig. 1:**
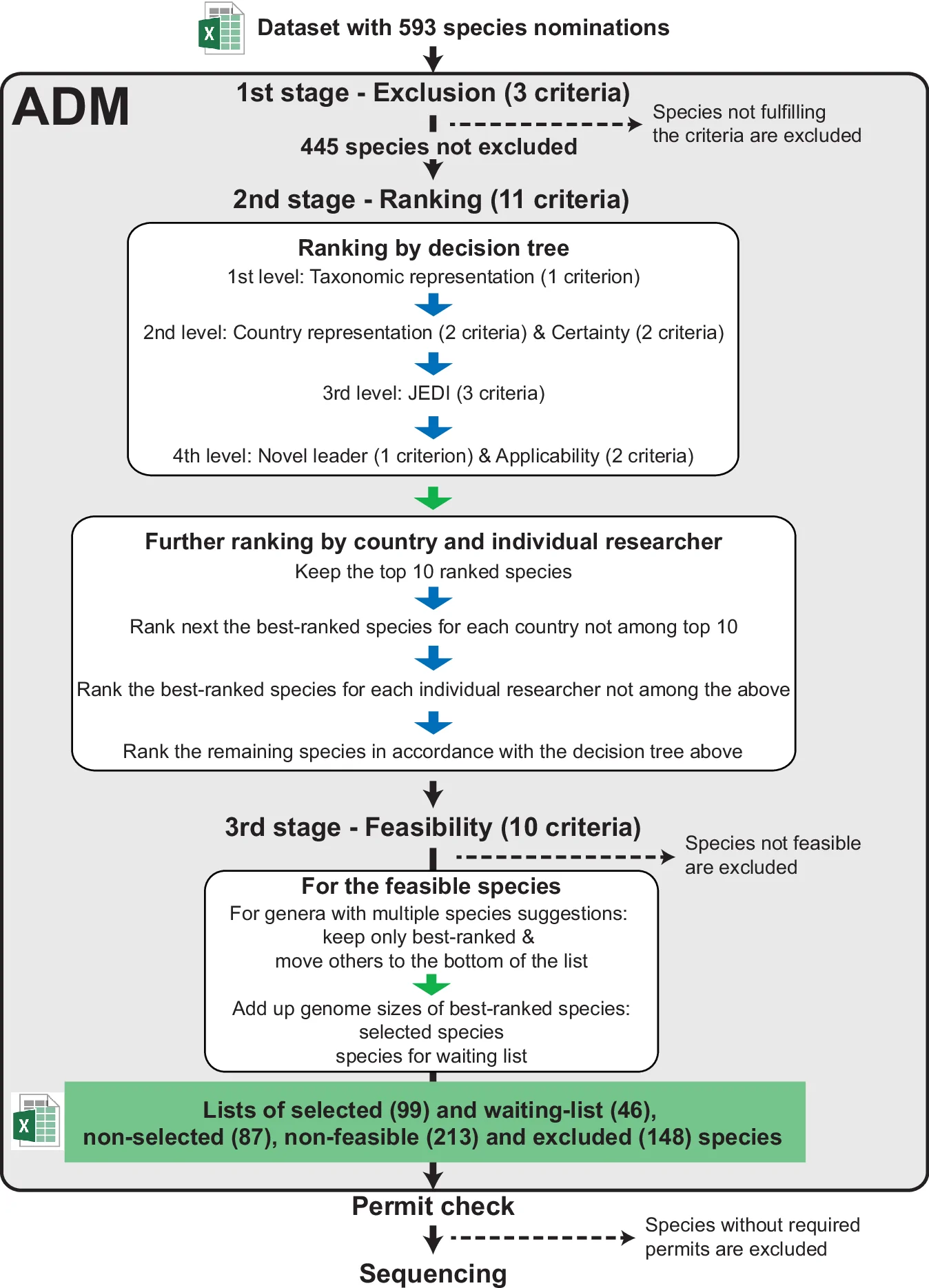
Flow-chart of the automated decision-making (ADM) process for species selection. The flow chart shows the process on how to select species for sequencing of reference genomes suggested by the community within the European Reference Genome Atlas (ERGA) stream of the Biodiversity Genomics Europe (BGE) project. The whole ADM process itself is highlighted by the gray box and subparts by white boxes. JEDI = Justice, Equity, Diversity, and Inclusion.

The species selection process implemented in the BGE project was a three-stage ADM process (Fig. 1) that comprised (1) an exclusion stage, (2) a general ranking stage employing a decision-tree and additional ranking for country and individual researcher representation, and (3) a feasibility check followed by an additional adjustment for genera for which multiple species had been nominated. The result of the ADM process consisted of different lists including the one of selected species and a waiting list.

The ranking process was based on a total of 24 criteria (Table 1 and Supplementary Table 1). Besides the selection criteria of the pilot project of ERGA^10^, additional criteria were suggested by the ERGA community and refined by the authors. This resulted in 45 different criteria, of which 24 were considered to be necessary for the selection process. These 24 criteria were sorted into the three stages of the species selection process. Three criteria were placed at the “Exclusion” stage. Eleven criteria were put at the second stage, “Ranking”, and used for the species ranking process. The third stage, “Feasibility”, consisted of 10 criteria. The information for all 24 criteria was retrieved from the questionnaire mentioned above. Hence, the gathering of the information was essentially trust-based on the self-assessment of the nominator. No missing data was allowed for these criteria.

**Table 1:** Individual ranking criteria of the first three stages of species ranking

Criterion	Threshold (stage 1 and 3)	Scale
	Category (stage 2)	
*Stage 1 - Exclusion*		
Availability of published reference genomes	None published	binary (yes/no)
Redundancy	Nonredundant	binary (yes/no)
Availability of voucher specimen	Voucher available	binary (yes/no)
*Stage 2 - Ranking*		
Certainty of species identification (1)	Certainty	Ordinal
Type locality (2)	Certainty	Ordinal
Taxonomic representation on Tree of Life (3)	Taxonomic representation	Ordinal
A minimum of one species per country (4)	Country representation	binary (1/ > 1)
Countries with fewer genomic resources (5)	Country representation	binary (yes/no)
Novel sample coordinator (6)	Novel leader	binary (yes/no)
Gender of researcher or gender balance of team (7)	JEDI	Ordinal
Researcher of underrepresented minority (8)	JEDI	Ordinal
Diversity & inclusiveness of team (9)	JEDI	Ordinal
Application of the genomes (10)	Applicability	binary (<1year/≥1 year)
Reach/breadth of community (11)	Applicability	binary (1–2 stakeholders/ >2 stakeholders)
*Stage 3 - Feasibility*		
Genome size	<6 Gb total haploid	binary (yes/no)
Sample availability	Available	binary (yes/no)
Species abundance and access	Abundant and easily accessible	binary (yes/no)
Number of samples and sampling across tissues	Multiple samples per specimen or specimens	binary (yes/no)
Size of sample	> Drosophila size or 100,000 nucleated cells	binary (yes/no)
Method of preservation	Snap frozen in liquid nitrogen or on dry ice	binary (yes/no)
Sample preserved suitable for Hi-C	Snap frozen in liquid nitrogen or on dry ice	binary (yes/no)
Time elapsed from death to preservation	Preserved in <5 min	binary (yes/no)
Sample preserved suitable for transcriptome sequencing	Snap frozen in liquid nitrogen or on dry ice	binary (yes/no)
Storage of sample	Validated cold chain of −70 °C	binary (yes/no)

For stage 2, numbers behind the criterion provide the ID used for this criterion in the explanation of the weighing schemes (see in “Weighting scheme of each category” in the Supplementary Information). Supplementary Table 1 provides more detailed information on the individual criteria, including a rationale for each.

Three criteria at the first stage, Species exclusion, needed to be fulfilled for a species to proceed to stage 2. If any of them were not fulfilled, the species was excluded. These specific criteria were “Availability of published reference genomes”, “Redundancy”, and “Availability of voucher specimen” (Table 1 and Supplementary Table 1, please also see “Individual selection criteria” in the Supplementary Information for details). At this stage, 148 species were excluded, and 445 species moved forward to stage 2 (Fig. 1).

In the second stage, species ranking, all species were first ranked using a decision model based on 11 criteria (Table 1 and Supplementary Table 1). Information for those was obtained from the sample providers when they nominated the species. These 11 criteria were grouped into six categories to make the process easier. Moreover, some of the individual criteria assessed different aspects of the same topic. The six categories were: “Taxonomic representation” (1 criterion; “Taxonomic representation on Tree of Life”), “Certainty” (2 criteria; “Certainty of species identification” and “Type locality”), “Country representation” (2 criteria; “A minimum of one species per country” and “Countries with fewer genomic resources”), “Justice, Equity, Diversity, and Inclusion (JEDI)” (3 criteria; “Gender of researcher or gender balance of team”, “Researcher of underrepresented minority” and “Diversity & inclusiveness of team”), “Novel leader” (1 criterion; “Novel sample coordinator”), and “Applicability” (2 criteria; “Application of the genomes” and “Reach/breadth of community”).

A weighting scheme was assigned to each category (explained in detail in “Weighting scheme of each category” in the Supplementary Information). In brief, the scheme of the category “Taxonomic representation”, ranging from genus to phylum level (and beyond), applied weights from 1–5, with higher weights for higher taxonomic units. In the one of “Certainty”, weights of 1, 2, 5 and 6 depended on how close to the type locality the sample was collected and how certain the species identification could be accomplished. Higher weights were given to species with higher taxonomic certainty. In the category “Country representation”, one criterion contributed to the knowledge transfer and hence was linked to the country of the sample coordinator. The other criterion contributed to an equal representation of species from across Europe and is accordingly connected to the collection site. This meant that one species could represent two countries in this category. Weights of 1, 4 and 5 were applied, with higher weights for countries from the list of EU Widening countries and the single species being nominated from the country. In the category “JEDI”, weights of 1–5 were applied depending on the sample coordinator’s gender, minority background, and the inclusiveness of the genome team, with higher weights assigned to increase diversity and inclusiveness. The category “Novel leader” used weights of 0 and 2 to reflect if the sample coordinator has already gotten a species sequenced via BGE/ERGA resources. Finally, the category “Applicability” had weights of 0-2 depending on how quickly and broadly the reference genome could be applied outside the nominating group.

Concerning the selection model of the ADM process, eight different ranking models were initially developed and tested (for more details on this step of the process, please see “Development of different prioritization models” in the Supplementary Information). Seven of these were variations of the final model presented below. These explored different combinations of “Certainty” and “Country representation” and of “Novel leader” and “Applicability” at different levels of a decision tree. The eighth model did not implement a decision tree but summed up the weights of all categories equally and ranked the species based on this sum. To explore the effect of the different models on the selection process and whether they generated the expected outcome, the models were applied to simulated and empirical data. For a detailed description of the procedures and results, please see “Simulation studies” and “Empirical data” in the Supplementary Information.

For the simulated data, three probability distributions for the weighting scheme of each category were used to randomly assign a weight for each category and simulated species. In one distribution, all weights were drawn with equal probability, and in the other two, the probability was skewed either towards low or high weights being more probable. All possible combinations of these probability distributions were generated, resulting in 729 (3^6^) combinations. For each combination, 100 datasets with 1,000 species were generated, resulting in 72,900 simulated datasets. The eight models described above were applied to each dataset to select either the top 100, 200, or 300 species, resulting in 218,700 sets of selected species. For each dataset, model, and top-selected species, an enrichment factor was calculated for each category. The enrichment factor measures the relative enrichment of a category in the top-selected species in relation to all species as the difference of the mean weight values of the top-selected species to all 1,000 species set in relation to the maximum possible difference. Hence, it was a relative measurement of the maximally possible positive enrichment of a category in the dataset with a maximal value of 1. Negative values were not bound, indicating a lower representation in the top-selected species.

**Fig. 2:**
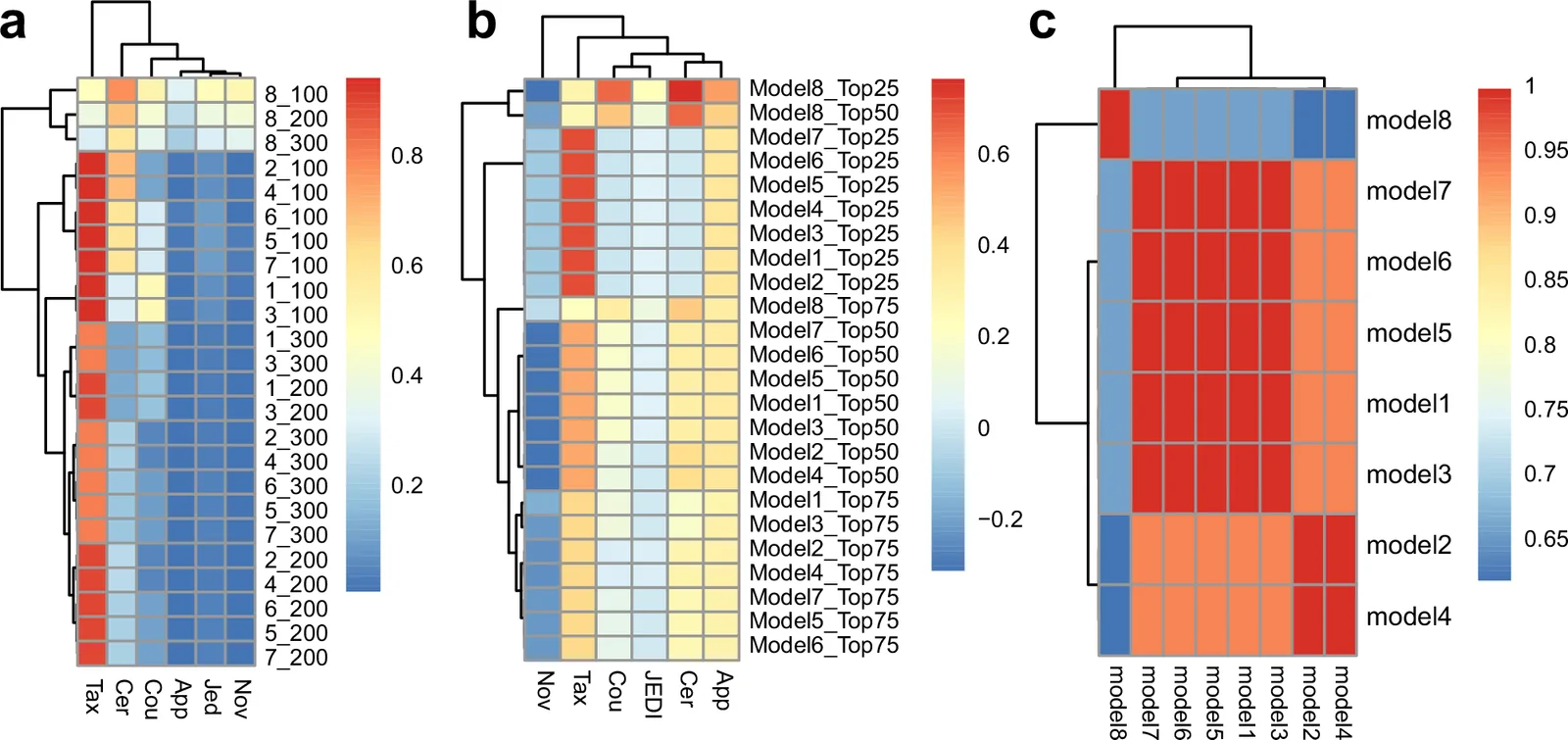
Impact of different possible models of the automated decision-making (ADM) process on the species selection. **a** Heatmap of simulation studies of the enrichment of the different categories given the model (first number, 1–8) and number of selected species (second number, 100, 200, or 300). **b** Heatmap of the empirical data of the enrichment factor of the different categories given the model (first number, 1–8) and number of selected species (second number, 25, 50, or 75). **c** Heatmap of the empirical data of the intersection between the 50 species selected by the eight different models. The value of the enrichment factor or the degree of overlap is indicated by bar at the right of each panel. App = Applicability, Cer = Certainty, Cou = Country representation, Jed = JEDI, Nov = Novel leader, Tax = Taxon representation.

A heatmap of the mean enrichment factor across the 729 combinations for each model and number of top-selected species for each category showed that model #8 is set apart from all other models (Fig. 2A). Moreover, all categories are more or less equally enriched, with the strongest enrichment in “Certainty” and the least in “Applicability”. Among the seven other models, the next split is between the selection of only 100 out of 1,000 species (10%). The models #1 and #3 emphasizing “Country representation” over “Certainty” in the species selection were thereby placed a bit closer to the other numbers of top-selected species than the other five. The major difference between these was that “Country representation” was more strongly enriched than “Certainty”, while it was the other way around in the models #2, #4-7. Moreover, in the models #5-7, which assessed both categories together, “Certainty” had a stronger influence than “Country representation”. Also, for 200 and 300 selected species, the models #1 and #3 were set apart from the other five, but less prominently. Generally, a similar pattern occurs as with the 100 selected species. In summary, models #1 and #3, #2 and #4, as well as #5–7 had no strong differences, while model #8 was set apart by having less emphasis on the “Taxonomic representation”.

For the empirical data, we used the 230 suggested species of the first round of suggestions by the community that passed the exclusion stage. Applying the eight different models to the dataset, we roughly selected 10% (25), 20% (50) and 30% (75) of the species, calculated the enrichment factor as above and also determined the intersection of the selected species between the models with the same number of selected species (i.e., for 25, 50 or 75 species).

As in the simulation data, model #8 was different from all other models (Fig. 2B). Within model #8, the differences were more pronounced with fewer species selected. The strongest enrichment was “Certainty”, followed by “Country representation”, and “Applicability”. While “JEDI” and “Taxon representation” had a low enrichment or none, “Novel leader” had negative values. In the case of the other seven models, the separation was between the number of selected species. Within the 25 selected species, there was no difference between the models. “Taxon representation” was strongly enriched, and “Applicability” was intermediate, while all others were not enriched. Selecting 50 species, enrichment of “Taxon representation” was not as strong, while enrichment of “Certainty”, “Country representation”, and “Applicability” was increased to intermediate values. “JEDI” showed no enrichment, while “Novel leader” had strong negative values. Moreover, models #2 and #4 were slightly different from the other five. Finally, the picture with 75 species looked similar, but for “Taxon representation” and “Novel leader” the values were not so strong any longer, and the differences of models #2 and #4, as well as #1 and #3, were a bit more pronounced.

Looking at the intersections of the 50 selected species showed that the overlap of selected species between the models was high, with equal to or more than 62% of species shared among any pair of models (Fig. 2C). Nonetheless, three groups could be recognized. One contained again only model #8. The other ones are models #2 and #4, which selected the same species. The last group contained the remaining five models, which also selected the same species. The similarity in species selection between these two last groups was also high, with 94%. Hence, like with the simulation studies, the similarity between the models #1-7 was high.

**Fig. 3:**
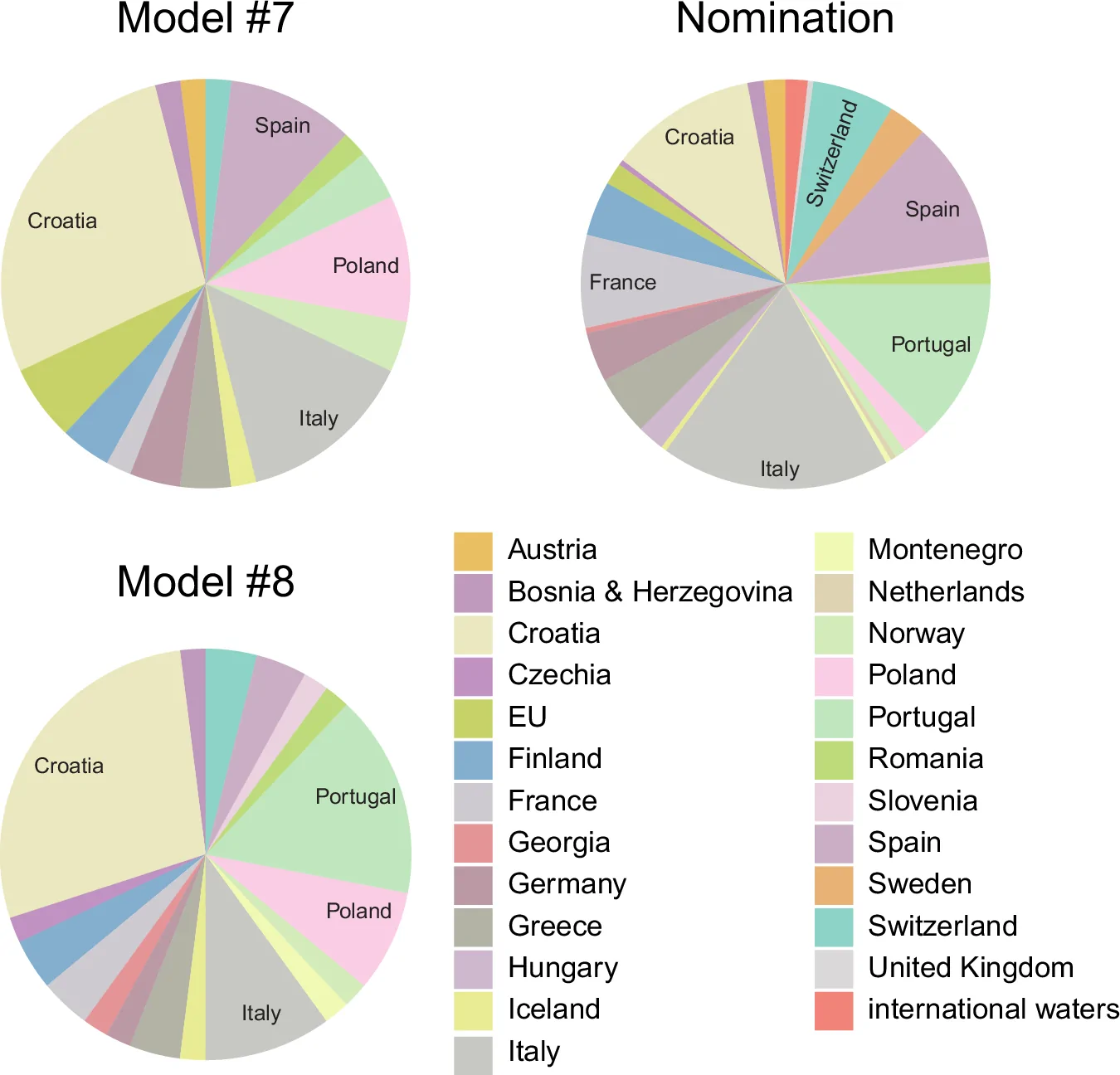
Representation of the countries where the species are to be collected. The representation is given for all nominated species, and the 50 selected species using models #7 or #8 in the test with empirical data. The color code for the countries is indicated in the lower right corner, and countries with higher representation are also labeled in the pie charts directly.

However, a more detailed investigation of the empirical data also showed that the country representation after the decision model was biased towards certain countries, independent of the applied model (Fig. 3). For example, Croatia was strongly represented and relatively more than among the nominating countries. Similarly, Poland and Italy were also highly represented. The original models also introduced a strong bias towards individual researchers as they had predominantly suggested species from underrepresented taxonomic groups (for more details, see “Additional step for prioritization” in Supplementary Information).

Given the results from the simulated and empirical studies, a final decision by the ERGA council was made on two of the eight models tested (i.e., models #7 and #8) and additional rounds of ranking (for more details, see “Decisions by the ERGA council” in the Supplementary Information). Model #7 was elected. The final model, hence, comprised four decision levels. The first level of ranking was based on the category “Taxonomic representation”, as this was regarded as the most important category by both the larger scientific community and the BGE project (Fig. 1). Hence, species with a higher score in this category were ranked above those with a lower score. Species with the same score in this category were then ranked amongst them based on the combined categories “Certainty” and “Country representation”. The scores for these two categories were summed for each species. Then the species were ranked based on the sum. All species that had the same scores in the two higher levels were ranked at the third level based on the category “JEDI”. Finally, all species that had the same scores in the three top levels were ranked at the last level based on the summed scores of the categories of “Novel leader” and “Applicability”.

Additionally, after the ranking by the decision tree, additional rounds of ranking were conducted (Fig. 1). The ranking of the top10 species was left unchanged. Next, the best-ranked species of each country that was not included in the top10 species was ranked after the top10 species. The order of ranking within these best-ranked species by country was based on the ranking by the decision tree. The country of interest in this process was the country of the collection site and not the country of the institution of the sample coordinator. This addition aimed to achieve a better representation of species from different European countries among the selected species. After this country-based ranking, the best-ranked species of each researcher, suggesting a species that was not included in the top10 species or the best-ranked country species, was ranked after these species. The procedure was the same as for the country ranking. This ranking aimed to distribute the benefits of the BGE project as widely as possible across the scientific community in Europe. Finally, the remaining species were ranked following the ranking from the decision model. The ranked list of all species after these processes was then transferred to the next stage. No species were excluded during the ranking stage.

The third stage, feasibility, consisted of 10 criteria that assessed whether a suggested species was feasible to be sequenced, given the available funding, current methodological capabilities^11^, and sample availability (Table 1, Supplementary Information). The feasibility thresholds were determined together with the sequencing centers. Species failing any of the feasibility criteria were flagged as non-feasible and removed.

After the feasibility check, a final round of ranking was conducted. A few genera were found to be represented by more than one species, which was assessed as not being in agreement with the goal to accomplish a broad taxonomic representation of the eukaryotic biodiversity. Accordingly, the same principle as for the best-ranked species for countries and individual researchers was applied to these genera. The best-ranked species of each genus was kept at its position in the ranking order; all other species were moved to the bottom of the list. Among all the species across the different genera that were placed at the bottom of the list, the same ranking order was kept as it had been in the original list. The final ranking produced a ranked list of feasible and non-excluded species. From this list, the top-ranked species were selected for sequencing, given the limit imposed by the sequencing capacity, plus additional species for a waiting list. In our case, we applied 50% of the sequencing capacity as the threshold for the waiting list. Hence, the selection process until stage 3 generated lists of feasible species (selected, on waiting list, or non-selected species), non-feasible species, and excluded species (due to lack of a voucher specimen, the availability of a reference genome, or the species being targeted by another ongoing reference-genome project).

The check of all necessary legal documentation (“Check of permits”) was placed before the actual sequencing. The legal responsibility that all permits necessary to access the sample for genome generation and genome data release had been obtained, was with the sample provider (usually the researcher nominating the species). Before the sample could be shipped to the appointed sequencing center, the sample provider had to also submit a standardized set of metadata to BGE^10,21^. Upon submission, the sample coordinator had to upload all necessary permits for the sample. Given our experience from the ERGA pilot project^10^, this crucial part was deliberately introduced late in the process as the package of necessary permits may depend on the attributed sequencing centre and the country it is based in (e.g., export and import permits). Hence, they were only provided at this stage of the process. Even for international agreements, such as the Convention on International Trade in Endangered Species of Wild Fauna and Flora (CITES) permits or Access Benefit Sharing (ABS) contracts as part of the Nagoya process, the landscape of their relevant national implementations is quite complex and challenging due to the bureaucratic idiosyncrasy of each country. For example, some European countries are quite flexible, while others are more restrictive^10^. Additionally, in some countries, permits or contracts are only issued in the native language, making an upfront implementation in an ADM more challenging.

### Effect of the selection process on the final species ranking

**Fig. 4:**
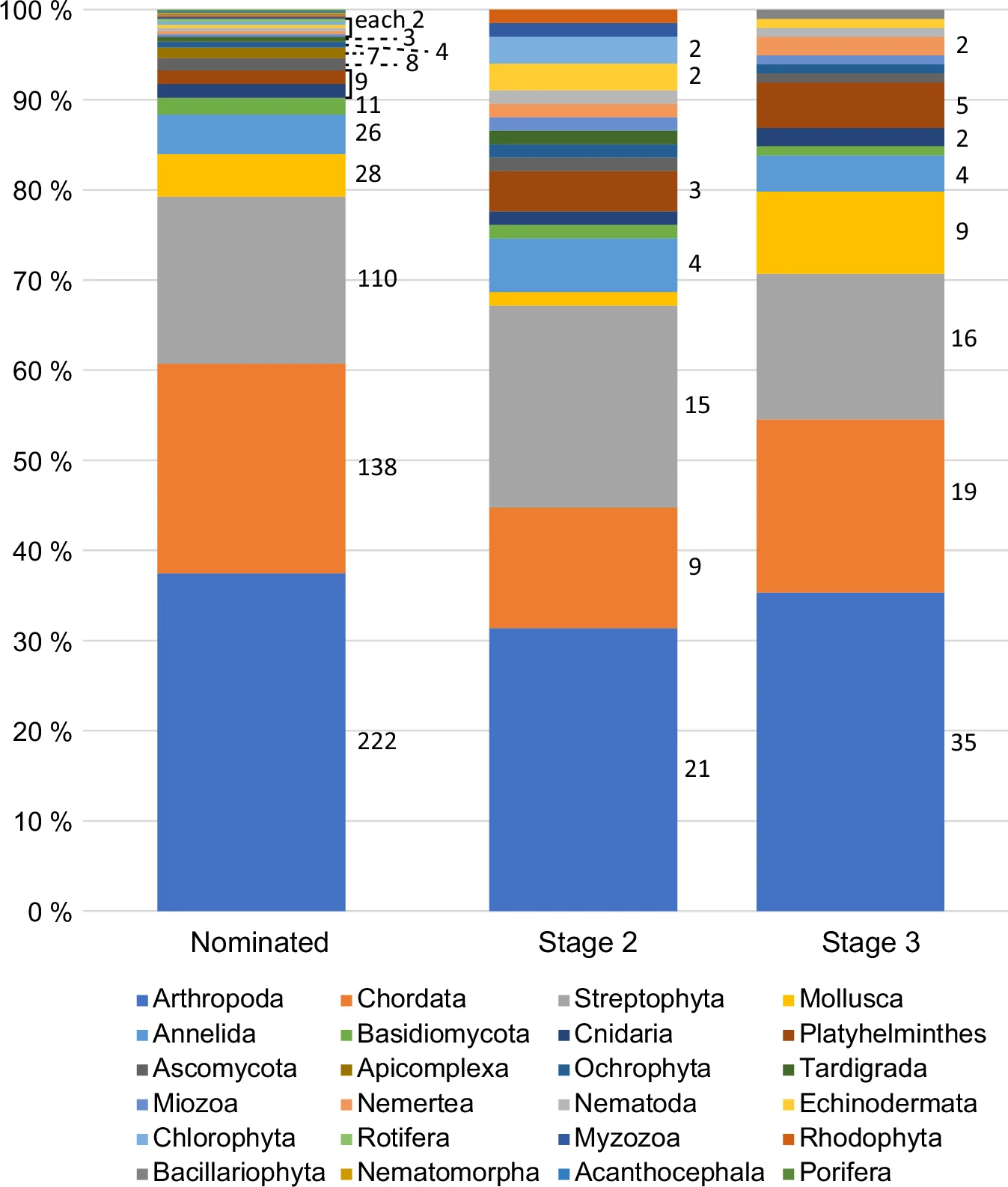
Phylum representation at three stages of the ADM process using the final model #7. The presentation of the phyla is given for three stages, from left to right: all 593 nominated species, before the 1st stage (exclusion); the 67 species that would have been selected after the decision model at the 2nd stage (ranking); the 99 species that passed the feasibility check and were finally selected for the empirical data. The taxonomy follows European Nucleotide Archive (ENA). The absolute number of species is shown to the right when larger than 1. The color code for the different phyla is provided below the stacked histogram.

**Fig. 5:**
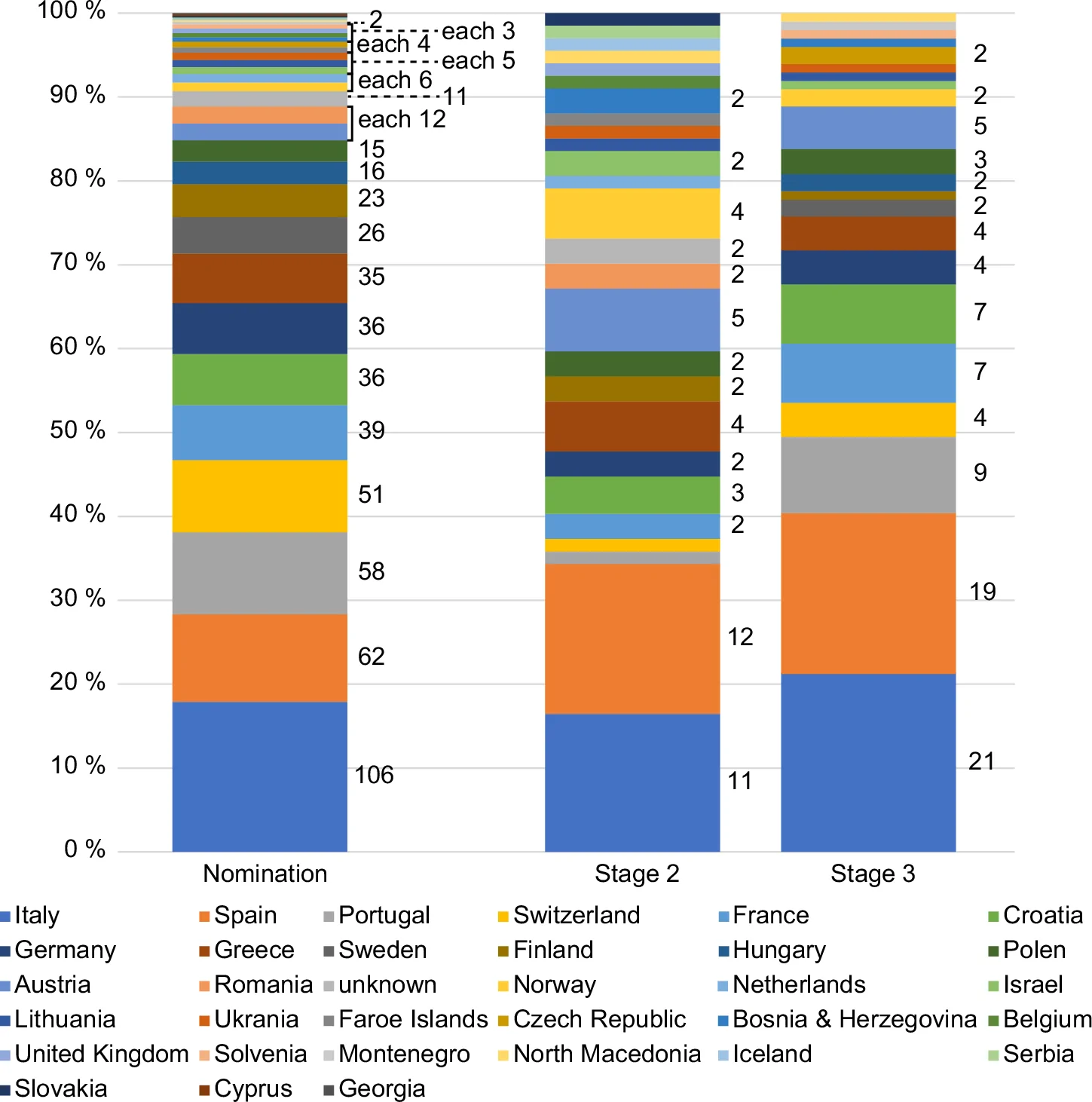
Country representation at three stages of the ADM process using the final model #7. The presentation of the countries is given for three stages, from left to right: all 593 nominated species, before 1st stage (exclusion); the 67 species that would have been selected that would have been selected after the decision model at the 2nd stage (ranking); the 99 species that passed the feasibility check and were finally selected for the empirical data. The color code for the different countries is provided below the stacked histogram. The number of species is shown to the right when larger than 1.

As mentioned above, we thoroughly tested the selection process and the different possibilities at different stages. Here, we will only present the outcome of the entire ADM process (i.e., the finally selected species) in comparison to the pool of the nominated species and the species that would have been selected without applying stage 3 (Figs. 4 and 5). We will also briefly present the results of the feasibility check and the reasons why species were not feasible.

BGE funding allowed for the sequencing of 150 Gb in the task “T5.5 Critical biodiversity community sequencing”. If the selection process had been done without the feasibility check directly after stage 2, 67 species would have added up to 150 Gb and would hence have been selected. Adding up the predicted genome sizes of the ranked species after the feasibility check (stage 3), 99 species were finally selected. The lower number of species after stage 2 was due to the fact that this list would have included some species with large genomes, such as *Somniosus microcephalus* (Chordata) with a genome size of ~10 Gb, *Calotriton asper* (Chordata) of ~27 Gb, or *Apocalathium malmogiense* (Myzozoa) of ~30 Gb. These three species alone would have comprised about 45% of the total 150 Gb.

However, the increased number of species did not result in broader taxonomic representation. In contrast, the selected 99 species comprised 15 phyla of the originally 24 nominated phyla (Fig. 4). The 67 species at stage 2 would have comprised 18 phyla. In the feasibility check, the only species representing Chlorophyta, Rhodophyta, Myzozoa, and Tardigrada were excluded, while a representative of Bacillariophyta was added. The phyla Arthropoda (35 species instead of 21), Chordata (19 instead of 9), and especially Mollusca (9 instead of 1) benefited the most from the feasibility check. Finally, even though 148 out of the original 593 nominated species were excluded at stage 1, only one representative of Acanthocephala was removed.

Considering the phyla originally nominated with more than 10 species, Arthropoda and Annelida were roughly represented at the same percentage in the finally selected species as in the nominated species. Chordata, Streptophyta, and Basidiomycota were less well represented, as relatively more species had been nominated, while Mollusca benefited from the process. Additionally, Platyhelminthes was also more strongly represented in the final list. Without the feasibility check, Arthropoda, Chordata, and Mollusca would also have been less represented compared with the original nominations, with the effect being especially prominent for the latter two.

Concerning the countries, all nominated species comprised 32 countries (Fig. 5). The species from Cyprus was excluded at stage 1. Selecting species directly at stage 2 would have resulted in 25 countries. Hence, six countries would have dropped out at this stage (i.e., the Czech Republic, Georgia, Hungary, Montenegro, Sweden, and Slovenia). The actual species selection after the feasibility check reduced the number of selected countries to 22. Specifically, Belgium, the Faroe Islands, Georgia, Iceland, the Netherlands, Romania, Serbia, Slovakia, and the United Kingdom were excluded. Hence, the Czech Republic, Hungary, Montenegro, Sweden, and Slovenia were not excluded as would have happened at stage 2. Generally, the distribution across the other countries was relatively even after both stages, with 14 countries having more than one species considered. However, Spain, France, Croatia, Italy, Switzerland, Germany, and Portugal benefited from the feasibility check with a substantial increase in species coming from these countries. Hence, the spread became less even after the feasibility check.

The distribution of individual researchers among the selected 99 species was very even, with a total of 92 researchers providing species and only five with two or three species. A similar pattern was also found for the 67 species at stage 2. This was in contrast to all species, where several individuals nominated multiple species. Hence, the goal to distribute the generation of reference genomes across Europe at both the country and individual levels had been generally accomplished, even though it was not perfect at the country level.

Only 5% of the selected 99 species had known taxonomic problems, but on the other hand, only about 7% of the 445 species after stage 1 had known taxonomic problems. Similarly, the proportion of species collected at or close to the type locality was about 49%. This was slightly better than all species after stage 1, with 45%. The relative composition of the procedures applied to identify species was not substantially different between the species after stage 1 and the selected species. Nonetheless, identification procedures applying only one approach were generally reduced among the selected species, including those that only involved a taxonomic expert for the group. Among the selected species, most (~75%) applied more than one method of species identification. In contrast, among all nominated species, “only” ~64% applied more than one method.

With respect to the gender distribution, male researchers comprised about 49% across the species after stage 1, while this increased to 54% among the selected species. Similarly, researchers not disclosing their gender also increased among the selected species (~5% from ~3%). Accordingly, female and non-binary researchers were both less represented. The proportion of underrepresented minorities (2.5%) or persons who did not want to disclose it (6.5%) was small in the pool among all species after stage 1, but due to the process still increased slightly to 3.0% and 7.1%, respectively. Moreover, among the selected species, about 52% of the genome teams were purely scientific, while it was only 45% for all species after stage 1. Otherwise, the composition of the genome teams in the selected species was not strongly different from the nominations. Hence, concerning JEDI criteria, the ADM process did not enrich these criteria and sometimes had some degree of the opposite effect.

### General effect of the feasibility check at stage 3

A total of 213 species (from 445 species after stage 1) were considered not feasible (47.9%). Closer examination of the reasons for the exclusion of a species due to feasibility criteria (Table 2) revealed the following. It should be noted that a single species could be non-feasible by more than one criterion. For example, 6.1% of the species had a too small body size and could also not be preserved on dry ice or snap frozen. The exclusion of 21.4% of the 213 non-feasible species happened due to both biological and non-biological criteria.

**Table 2:** Exclusion based on individual feasibility criteria

Individual criterion	Count	Percentage
Genome size	46	21.6
Body size	47	22.1
Availability	42	19.7
Preservation	62	29.3
Processing time	45	21.3
Cold chain	58	27.4

Of the non-feasible species, 21.6% were excluded due to a genome size larger than 6 Gb, and 22.1% due to a sample size smaller than a *Drosophila* fly or less than 100,000 nucleated cells. 41.3% of the species were excluded because they fulfilled one or both criteria. 2.4% of the species had a small body size combined with a large genome. All of these species are challenging, even with new sequencing technologies. Moreover, 29.1% of the species were excluded exclusively due to small body size and/or large genome size.

Additionally, 19.7% of the species did not fulfill the criteria that they were already collected or easy to obtain (i.e., they were either common, widespread, and abundant or rare, but locally abundant). Within these 19.7%, 26.2% were also regarded as non-feasible by other criteria, but 73.8% of them, or 14.6% of all non-feasible species, were just challenging to collect. Hence, almost half (29.1% + 14.6% + 3.3% = 47.0%) of the species were considered not feasible purely due to biological properties.

Considering non-biological reasons, 45.3% of the species were considered not feasible due to criteria related to the sampling and sample processing. 29.3% of the species could not be snap frozen or frozen on dry ice for sample preservation. For 21.3% of the non-feasible species, it was not possible to preserve them within 5 min after their death, and for 27.2% it was not possible to maintain a strict cold chain at −70 °C. Of the non-feasible species, 31.6% were purely excluded because they could not properly be preserved or maintained. Hence, about one-third of the exclusions happened just due to non-biological criteria.

## Discussion

We have shown that automated decision-making (ADM) (i) worked on a large-scale biodiversity project, including community input at all levels, (ii) that feasibility and funding shaped the final set more than anything else, and (iii) that representation improved in aspects but still reflected the original composition of the nomination pool. By our ADM process, we demonstrate an alternative to the more subjective selection of species for sequencing by an expert panel and/or governmental authorities^22^. Moreover, before a final decision on the ADM process is made, it can be tested using both simulated and empirical data to assess if the desired outcome is achieved.

For example, for the ERGA and BGE community, taxonomic representation across the tree of life was the most important factor, in line with the goals of EBP in general^2^. The next two important goals were a broad representation across the European scientific community (both on the country and individual level) as well as taxonomic certainty in the species identification. The former one is in line with the EU goals for knowledge transfer to the so-called widening countries^23^. The latter strengthens the goals of good taxonomic practice and reliable species identification as the foundation of biological science^24–28^. This is also reflected not only by the applied decision model but also by the additional ranking steps included in the process, which aim to increase the representation of taxa, countries, and individuals, as well as the requirement for specimen vouchers. Across both rounds of selection in the BGE project, the process generally accomplished these goals. However, both taxonomic and country representation were more strongly affected by the different stages of the process. While about 25% of species were excluded at stage 1, only one phylum and one country were no longer represented. Hence, stage 1 had little impact on the taxonomic and country representation. In contrast, both following stages had a stronger impact on both the taxonomic and the country representation. Nonetheless, both taxonomic and country representation were still relatively high and broad due to the ADM process and are not biased by subjective decisions. As we will show in the following, the inclusion and exclusion of phyla and countries can be essentially explained by the combination of feasibility, funding, and nomination.

The feasibility criteria and funding limitations on species selection had an effect on the species selection. Feasibility, either direct or indirect, severely affected which species were selected for genome sequencing and, as a consequence, conflicts with the EBP’s goal to sequence every eukaryotic species on Earth^2^. A large proportion of the BGE samples were omitted because they failed to meet one or several of the a priori defined feasibility criteria. Interestingly, even though substantially more species were selected after stage 3 than would have been after stage 2, the number of selected phyla reduced further instead of increasing due to the higher number of species. Several of the feasibility criteria are linked to features of the species, such as abundance, genome size, and size of the sample, and about half of the non-feasible species were excluded due to any of these biological criteria. These characteristics are also prone to have a systematic and phylogenetic bias; for example, meiofaunal species are not evenly distributed across the animal tree of life^29–31^, genome size is evenly not across the eukaryotic tree of life^32^ or protists pose challenges to genomics in several aspects^33^. Accordingly, the four phyla excluded due to the feasibility check had either too large genomes (Myzozoa), too small body size (Tardigrada), or were too difficult to sample (Chlorophyta, Rhodophyta). However, in the BGE data, we did not see a clear phylum-level pattern, but sample size and nominations limited what one can conclude. Final coverage largely tracked what was nominated per phylum. For example, only a few species of phyla with small body sizes, such as Tardigrada, Rotifera, or Nematoda, were nominated or none at all, like for Gnathostomulida, Gastrotricha, or several unicellular eukaryotic phyla. Hence, an implicit filtering might have already occurred at the nomination level, suggesting that we clearly and transparently communicated the feasibility criteria.

Nonetheless, enforcing upper or lower limits to these biological criteria may be valid for practical reasons, but it is also obviously in conflict with EBP’s goal to sequence every species on Earth^2^. It is therefore of pivotal importance that methods be developed or improved to reduce potentially systematic bias^11^. The accumulation of expertise in processing diverse samples in the lab has led to the development of improved ultra-low extraction protocols that can better handle smaller organisms with larger genomes^34–36^. Also, the development of specific protocols for different organism groups is helpful in this matter^11,37–39^.

The ever-decreasing costs of genome sequencing help to gradually lower the threshold for what is considered an unfeasibly large genome by genome sequencing consortia. Indirectly, the large genomes would have exerted a strong impact on the selection of species at stage 2 and hence on the species included. Due to the large genomes and the limited funding for total genome size, the inclusion of large genomes would have substantially reduced the number of species compared to the actual selection. This reduction in numbers would have a strong impact on the representation of countries. All of the six countries that would not have been considered at stage 2 would only have been included if the sequencing capacity had been three times higher. This effect is also highlighted by the fact that five of them were re-included in the final selection, which included the exclusion of these large genomes as non-feasible, among others, due to funding limitations.

Our results also identify the ability to snap-freeze and maintain a cold chain for the collected sample as a seriously limiting factor to the feasibility of samples for genome sequencing, as almost one-third of the species were considered not feasible due to these factors. The proportion of non-feasible species from EU widening countries among the non-feasible species is almost identical to the proportion of all species included in stage 2. In general, for country representation, the original number of species nominated and included in stage 2 had a much stronger impact. Countries, which were on the borderline of being included or not, had usually nominated less than 1% of all species. The exceptions were Sweden, Hungary, and Romania, with 2.5%, 2.5% and 1.8% nominated species. The former two were re-included at the feasibility check, while Romania dropped out. On the other hand, all countries benefiting from the feasibility check by an increased number of selected species had nominated 5-18% of all species. Perhaps unsurprisingly, as with taxonomic representation, the number of nominated species exerted a strong impact on the final country representation.

Nonetheless, the problem of maintaining a cold chain can disproportionately affect the selection of species occurring in remote locations, such as oceanic islands like the Azores, which also frequently harbor a high proportion of endemic biota, or the deep sea. An obvious solution is access to dry-shippers, i.e., shipping containers whose cooling elements work by absorbing liquid nitrogen with no risk of spillage, which allows them to be brought on regular public transport, including planes. Additionally, this can be avoided through careful logistic planning, e.g., by “bioblitz” excursions with intensive sampling of taxa within a remote location. Finally, the development of new laboratory methods and chemicals to preserve DNA at higher temperatures might counteract this type of bias.

In the following, we will discuss the applicability of ADM frameworks to other projects. The ADM process presented herein is tailored toward the subjective priorities of the BGE project and ERGA community, given the goals and funding framework of the project and the technical capacities of the participants. Objective criteria were selected accordingly in an inclusive and bottom-up procedure. Beyond the bespoke application of this decision-making tool to BGE, we provide a roadmap for developing such a tool for other applications. The specifications and implementation of the ADM are adaptable to each community or project. Ultimately, prioritization in the ADM process is based on human-made, pre-defined lists of criteria. How these criteria influence prioritization can be informed by a project’s goals, limitations, and capacities, such as available funding, taxonomic focus, and technological capacity. Accordingly, the output of a ranked list is inherently flexible so that selection and ranking thresholds can be adjusted to reflect desired priorities. We have also provided an example of how the outcome of an ADM can be tested using simulated and empirical data to evaluate its effectiveness towards overall project goals. Large-scale, complex decision-making challenges are ubiquitous given that resources are limited. Therefore, it is not only large-scale genome projects that may find utility in the methods presented herein, but we will focus on this type of application.

The ADM process comprised three stages. The number of stages is adaptable, though we regard all three as principally relevant for any large-scale genome project such as BGE. Towards the EBP mission of sequencing a reference genome for each eukaryotic species within 10 years, avoiding redundancy across different projects allows a critically effective use of resources. Hence, adopting similar exclusion criteria of redundancy might be sensible for other projects. Redundancy avoidance is also enhanced if the species is identified accurately, and proof of the identification can be provided by the presence of a voucher for the identification. This will ensure high certainty that the genome sequence belongs to the species it is supposed to represent and better guide future sequencing efforts. Conversely, depending on the capacity and access to scientific collections, the criterion for a specimen voucher could alternatively be moved to the ranking stage rather than the exclusion stage.

Concerning the ranking stage, taking into account the taxonomic distribution across the tree of life would benefit other genome projects in accomplishing the EBP mission. Applying such a taxonomic criterion would more quickly result in a better representation of genomic resources across the tree of life. Accordingly, it would contribute to closing the knowledge gaps in our understanding of the evolution and ecology of biodiversity on Earth and increase our knowledge of how to maintain and recover it. By contrast, in some regions or countries, funding can be restricted to specific priorities such as economically driven applications or generally require a stronger emphasis on applicability over fundamental research. The ranking procedure could then emphasize applicability more strongly than in our model. Other decision-making bodies or funding calls might support biodiversity for environmental reasons but limit funding to, for example, species or habitats of particular conservation concern, like red-listed species, pollinators, tropical forests, or marine environments. Regardless of the priorities of any given community or funding body, the criteria and weighting of the ADM process can be adjusted to meet these needs and align priorities with policy in an objective way.

Similarly, a variety of social criteria could be prioritized in the ranking model. We prioritized country and individual diversity to enhance representation of the ERGA community among those directly associated with nominating and providing samples for reference genomes, who presumably will accrue benefits from them. Knowledge transfer and capacity building activities associated with a species are other potential criteria that can broaden participation in biodiversity genomics and contribute to non-monetary benefit sharing of genetic resources^40^. Cooperation and co-creation with relevant communities (e.g., local, research, taxonomic) can also be prioritized to enhance the scientific excellence and ethical soundness of research projects^41^.

Feasibility checks are necessary for genome projects to avoid wasting limited resources. We applied strict and absolute thresholds to the feasibility criteria. A nominated species had to pass each threshold to be selected. Given the wide variation in technical resources globally (e.g., access to infrastructure, technical expertise), thresholds for feasibility criteria can be customized for a community’s technical capabilities and limitations, either by adjusting threshold levels or by reducing the number of criteria that need to be fulfilled. Another option is to include feasibility criteria within the ranking model as additional ranking criteria with corresponding weights. For example, in regions or countries with higher average temperatures, maintaining a cold chain at -70°C might be particularly challenging. One could lend higher priority to providers capable of fulfilling this criterion while not excluding providers unable to fulfill it. However, this requires resources and expertise for handling suboptimal samples or developing adjusted protocols for such samples. These complex trade-offs highlight the importance of thorough discussion and weighing of different parameters in the prioritization process prior to sampling. Therefore, the design of the ADM process is greatly benefitted by broad involvement of the relevant community (see next section).

Feasibility checks can reveal obstacles still in the way of generating reference genomes representing certain species or regions. Rejected species can point out limitations in sequencing technologies, such as genome or sample size, or in sampling procedures, such as snap-freezing and cold chain maintenance. These insights can guide research and development efforts and justify improvements in infrastructure and expertise.

The procedures present here allow also for a strong community engagement in biodiversity genomics. A strength of the ADM process and its implementation in the BGE project is its explicit, bottom-up design. The general community nominated all of the species and decided on the ranking criteria and mechanism applied to the species. This effort ensured that this ADM process is less subjective, based upon the needs of the ERGA community at large, and beyond the BGE project, and that communication about the different criteria and their application was transparent from the very beginning. It can be seen that the BGE efforts of generating reference genomes are widely recognized among individual researchers across Europe, as there were almost as many individual researchers as possible species (i.e., 92 for 99). Moreover, the ability to test the outcomes a priori allowed adjustment of the process early on, minimizing the risk of unintentional outcomes in contradiction to the desired main goals.

Despite challenges with feasibility criteria and funding limitations, the ADM process ensured a relatively high taxonomic and country representation, with 62.5% and 68.8% of the nominated phyla and countries being considered. However, unevenness persisted due to strong biases in the nominated species with respect to a phylum or a country. Hence, while the ADM process was able to level the playing field to some degree, it still could not solve the initial bias in preferences. This could also be considered a strength of the approach, as the nomination process also shows where the majority of the community sees a need for reference genomes. Alternatively, independent of the limitations imposed by the feasibility criteria, it could also indicate that more efforts are needed to engage the scientific community from the underrepresented countries and phyla.

Finally, criteria related to principles of social justice, equity, diversity, and inclusion are core principles of ERGA (https://www.erga-biodiversity.eu/social-justice). Even though they were not the main goal of this ADM process, one aim was, nonetheless, to improve JEDI aspects in the distribution of the BGE resources across Europe. However, due to the low weight assigned to these criteria in the decision model and the absence of additional steps addressing them, no enrichment in these factors was achieved. In some cases, like for gender balance, the opposite occurred. This is because JEDI criteria only became relevant for very few species; those on the borderline of being included (if at all), and only when the species were identical in the previous two levels of the model. Hence, its overall impact is limited, and accordingly, any intrinsic bias present in the species scoring highest in the first two levels is transgressed through the process. The reasons for this can be multiple. For example, concerning gender balance, male researchers are slightly less represented among the non-feasible species, with 48% instead of 49%, slightly favoring them for the final list of selected species. Similarly, male researchers were present from 26 countries and female researchers from 22 countries, considering all species at stage 2. Given the goal to increase country representation among the selected species, male researchers are present from 18 countries and female researchers from 13 countries. Hence, given the low sample size of 99 species, these small intrinsic biases with gender representation among countries and feasible species in the pool of nominated species can explain the slight relative increase in male researchers. Again, a balanced representation already in the nomination pool seems more crucial here than tweaking the ADM process itself. This can be accomplished in the future by targeted outreach in underrepresented groups and countries encouraging their participation and lowering barriers to nominating species with an even better information flow.

There are future directions of ADMs in biodiversity genomics that can also be considered. Herein, we applied a fixed ranking list with only two rounds of nomination. In addition to the suggestions already made, for future (and ongoing) biodiversity projects, it is possible to envision a dynamic list of candidate species. The ranking could run on a rolling basis as new species come in. Species will get added to the initial candidate list, and the species ranking will be repeated each time. A process with repeated (or continuous) species additions will be more similar to a triage, i.e., a *prioritization* process^17^, rather than a *ranking* process. Specifically, species that have started being processed will not be stopped, but newly added species may be prioritized over species that were already on the list. This could be the situation if, for instance, the project aims at acquiring species by means of subsequent bioblitzes, i.e., intensive sampling events where numerous species are collected at once and repeatedly throughout the project. This would ensure a more effective usage of the capacity in line with the set goals. For example, important gaps in taxonomic representation could be filled much faster by such an approach, as species filling such gaps would instantaneously be prioritized and processed.

In principle, such an approach could already be conducted with the ADM process presented here. New species are added to the initial list, and species already being processed are removed from the list. Additionally, scores such as taxonomic representation are adjusted for all species in light of new information. For example, taxonomic representation of a species could be set to a lower taxonomic level if a species of the same higher taxonomic level is already being processed within the project or by other projects. The ADM itself (an R script) takes only a few minutes, and the maintenance of the list is probably more time-demanding than the ADM.

With the BGE project, we show that involving the community in the nomination and selection of species is an efficient and equitable way to maximize and ensure diversity in sampling species for biodiversity studies. The lack of reduction in diversity in the two key ranking criteria highlighted here, i.e., taxonomic representation and country representation, demonstrates that the automated species ranking applied by BGE respected the diversity of needs from the broader ERGA community. These findings reflect the strength of our approach and its capacity to balance different and sometimes conflicting interests, which might be beneficial for other consortia to consider when designing similar selection processes, even if their specific goals require different ranking criteria.

## Methods

### Development of the model

The Sampling and Sample Processing (SSP) committee of the ERGA community developed and agreed upon a set of selection criteria (Table 1 and Supplementary Table 1) and placed these into six categories. For each category, weighing schemes were developed to allow for nuanced application of the criteria within each category (see Supplementary information). The importance of each category for the ADM was determined by a poll among the members of the committee. Given the results of the poll, the SSP developed eight models to reflect the uncertainties of the poll. Seven models implemented a decision tree. In all seven, the top level was the category “Taxonomic representation”. At the next two levels, the categories “Certainty” and “Country representation” followed in different orders or in combination. At the next level, followed the category “JEDI”. At the bottom levels, the categories “Novel leader” and “Applicability” occurred in different orders or in combinations. The eighth model was a special case of the decision tree. In this case, all categories were treated equally, and the weights were summed up. It essentially means that all six categories were assessed at one level. One should note that an implicit weighing was present due to the different weights (especially maximum weights) for each category. The eight models were tested using empirical and simulated data (see below). The results were summarized and the final decision about the ADM model was taken by a poll of the ERGA council.

### Analysis of simulated and empirical data

For the simulation studies, three different probability distributions for the weighing scheme of each category were implemented (Supplementary Fig. 2). These probability distributions were used to randomly assign a weight for each category and simulated species. In the first probability distribution, all weights were drawn with equal probability. In the second one, it was more likely that low weights were assigned, in the third one, higher weights. Then, all possible combinations of these probability distributions were generated. This resulted in 729 (3^6^) combinations. For each combination, 100 datasets with 1,000 species were generated by randomly assigning a weight for each category to a species, given the applied probability distribution. This resulted in 72,900 simulated datasets. The eight ADM models were applied to each of these datasets, and the top 100, 200 and 300 species were selected, given the ranking of each model.

For each dataset, model, and top-selected species, the enrichment factor for each category was calculated. The enrichment factor measured the relative enrichment of a category in the top-selected species in relation to all species using the following formula:

$$\frac{\overline{Weigh{t}_{Top}}-\overline{Weigh{t}_{All}}}{Ma{x}_{poss.}-\overline{Weigh{t}_{All}}}$$

With $\overline{Weigh{t}_{Top}}$ being the mean of the weight values of the top-selected species, $\overline{Weigh{t}_{All}}$ being the mean of the weight values of all 1000 species, and ${{Max}}_{{poss}.}$ being the maximally possible value for the category. Hence, it was a relative measurement of the maximally possible positive enrichment of a category in the dataset. Negative values are possible and indicate that the category is represented less strongly in the top-selected species. Independent of the dataset, the number of top-selected species and the category, positive values always have an upper limit of 1, while there is no equivalent general lower limit. To explore the 218,700 (3*72,900) results more comparatively, for each combination, model, and number of top-selected species, the mean enrichment factor across the 100 datasets generated for each combination of probability distributions was calculated. For each model and number of top-selected species, heatmaps combined with hierarchical clustering were estimated for the combinations versus the categories. Next, the mean across the 729 combinations for each model and number of top-selected species for each category was calculated. Of these, a heatmap combined with hierarchical clustering was generated for the models and number of top-selected species versus the categories.

For the empirical data, the data were generally analyzed similar to the simulated data. We top-selected roughly 10% (25), 20% (50) and 30% (75) of species and used the enrichment factor to assess the outcome for the eight models. Additionally, the intersection of species selected between the models within the cohort of selected species as well as the proportion of the different options of certain criteria before and after the selection were calculated.

## Supplementary information

Supplementary Information

## Data Availability

Datasets to test the script are available at: https://github.com/torstenstruck/BGE_species_priorization.

## Code availability

The R script for the ADM process is available at: https://github.com/torstenstruck/BGE_species_priorization.
