# Supplementary material for: An automated decision-making procedure for ranking and selecting species in biodiversity projects
Source: NPJ Biodivers. 2026 May 30;5:30. doi: 10.1038/s44185-026-00140-x (PMC13626951; doi:10.1038/s44185-026-00140-x)
Supplement: Supplementary file 1 [file 44185_2026_140_MOESM1_ESM.pdf]

Supplementary Information

Contents

Supplementary Information 1
Detailed description of the selection criteria 2
Development of different prioritization models 5
Weighing scheme of each category 8
Weighing scheme of category “Taxonomic representation” 8
Weighing scheme of category “Certainty” 8
Weighing scheme of category “Country representation” 8
Weighing scheme of category “JEDI” 9
Weighing scheme of category “Novel leader” 10
Weighing scheme of category “Applicability” 10
Simulation studies 10
Empirical data 13
Additional step for prioritization 22
Decisions by the ERGA council 26
Questionnaire for the nomination in round #2 27

Detailed description of the selection criteria

Supplementary Table 1: List of the individual criteria at each stage including an explanation of the criterion and the applied threshold if applicable. For stage 2, numbers behind the criterion provide the ID used for this criterion in the explanation of the weighing schemes (see in “Weighting scheme of each category” in the Supplementary Information).

Criterion	Explanation	Threshold
	Stage 1 - No-go criteria	
Availability of published reference genomes	A genome is considered a reference genome given the EBP criteria, specifically, contig N50 > 1 Mb, scaffoldN50 > 10 Mb (with exceptions where chromosomal N50 < 10 Mb), chromosomal, and BUSCO > 90%.	Such species are excluded.
Redundancy	Species for which a high-quality genome that meets the EBP minimum standard is in process of being generated, e.g., by other consortia such as DToL or EBP-Nor.	Such species are excluded.
Availability of voucher specimen	The voucher specimen should be ideally what is left of the specimen after extractions for DNA, RNA and Hi-C. These remains should enable morphological identification. For small species that are completely consumed in the extraction, a proxy voucher from the same clone or population (collection event), and if relevant of the same sex, should be provided. A photo of the original specimen should be provided. In very rare circumstances (e.g., for endangered species) and for some taxa (e.g., gastrotrichs), this picture can also suffice as a digital voucher in the absence of a physical voucher.	Species without such a voucher are excluded.
	Stage 2. Species ranking criteria.	
Certainty of species identification (1)	As the goal of EBP is to sequence reference genomes for all eukaryotic species, the species need to be identified with certainty. The certainty of the identification needs to be indicated (e.g., known taxonomic problems with the taxon, cryptic species complex) and the procedure of identification (e.g., four-eyes principle, available relevant keys for the region, taxonomic expert named, barcode provided beforehand) be provided.	Priority is given to species that have been identified with a high degree of certainty.
Type locality (2)	Specimens close to the type locality are taxonomically preferable as they are more likely to be genetically close to the type specimen of the species and hence its name bearer.	It is advantageous if the species can be collected at least close to the type locality.
Taxonomic representation on Tree of Life (3)	EBP has as its first goal to sample and generate reference genomes for family representatives and BGE has also the goal to provide a broad representation of biodiversity. For some species,	Priority will be given to species representing higher taxonomic groups with no or few reference genomes. For incertae sedis

	the taxonomic level of relationship to the closest relative with a sequenced reference genome is, for example, within the same genus, family, order or phylum.	taxa, the species shall be placed at the highest known certain taxonomic placement of the species and the taxonomic level be assessed based on that placement.
A minimum of one species per country (4)	ERGA as a European-wide society and BGE as an EU consortium aim at generating reference genomes and increasing competence in genomic methods for the whole of Europe.	Biodiversity shall be sampled across Europe with at least one species per country, according to the sample site. To ensure inclusivity a requirement for the fulfilment of this criterion is that the genome team includes people from the country where the sample is to be collected.
Countries with fewer genomic resources (5)	Countries with low generation capacity of high-quality genomes (e.g., below a certain %GDP investment in R&D, lack of appropriate sequencing facilities or bioinformatics infrastructure, fewer genomic publications) should be prioritised over countries with greater capacities. The EU has the same inclusivity goals in its COST actions with the Inclusiveness Target Countries (ITC) and with the Widening Countries in the Horizon Europe program.	The Widening country list is used for prioritisation in this criterion.
Novel sample coordinator (6)	The goal of BGE/ERGA is also to ensure that the benefit of generating reference genomes and competence building is not only distributed across the European countries, but also across individual researchers. This criterion is similar to the incentive the EU-Assemble and EU-Assemble+ programs had.	Proposers who have not already gotten a species sequenced via BGE resources in a leading role are prioritised.
Gender of researcher or gender balance of team (7)	ERGA aims to follow the Justice, Equity, Diversity, Inclusion (JEDI) principles and increase the inclusiveness and diversity of the genome teams. ERGA wants to empower especially gender diversity in leading roles of genome teams.	Female sample coordinators get precedence.
Researcher of underrepresented minority (8)	Increasing diversity does not only apply to gender, but also to other equity-deserving (also known as 'underrepresented') groups in the genomics community. Such groups can be for example Black people, Indigenous people, People of Colour, Roma, and refugees.	Sample coordinators from such groups get precedence.
Diversity & inclusiveness of team (9)	Diversity and inclusiveness should not only be represented in the leadership position, but also in the genome team itself. Moreover, BGE/ERGA aims to broaden the impact of the generated genomes by including non-scientific stakeholders.	Genome teams having support from non-scientific interest groups or organisations (e.g., NGO, indigenous council) or representatives from these are prioritized. Similarly, priority is given to genome teams comprising

		disabled/handicapped people or citizen scientists.
Application of the genomes (10)	The generation of reference genomes should not be an aim by itself, but also benefit the larger scientific and non-scientific community. The faster such an impact can be generated the better for the visibility of the goals and sensibility of the EBP approach in general.	Genomes that shortly after publication will be used in research beyond the genome team are prioritised over ones with a longer time frame, as those genomes can be sequenced later.
Reach/breadth of community (11)	Besides a quick application of the reference genomes, it also is beneficial if the genomes are used by a variety of stakeholders and communities, increasing the reach and breadth of the application of genomic resources.	The more communities/stakeholders will benefit from the genome, the higher the priority.
	Stage 3. Feasibility criteria.	
Genome size	The project aims to sequence all species but recognizes that generation of reference genomes from species with very large genomes will consume significant shared resources and will limit the overall number of species sequenced. Moreover, very large genome sizes are still methodologically demanding with the amount of High Molecular Weight DNA needed.	Species with a haploid genome size above 6 Gb are regarded as not feasible and excluded.
Sample availability	Sample acquisition is a critical step in the process, and excessive delays will jeopardise delivery across the whole project. The project needs to deliver to its stated aims.	Species for which samples are already available with the collectors or can be easily collected are included.
Species abundance and access	The successful collection of species depends on its abundance and accessibility. The more abundant a species is and the more accessible (e.g., not in remote locations), the easier it is to collect.	If the proposed species still needs to be collected, only species were considered that are abundant and easily accessible.
Number of samples and sampling across tissues	The generation of reference genomes generally proceeds from samples of a single individual. Since some species have small specimens, generating all three kinds of data (long read, long range and transcriptome) can be difficult. In the case of small specimens, it is acceptable to sequence transcriptomes from secondary specimens. It is also possible, but less optimal, to sequence Hi-C long range data from secondary samples (even pooling several samples can be an option at this step).	Only species for which multiple specimens, with perhaps multiple samples per specimen are available, were included.
Size of sample	The intention is to assemble genomes from single individuals, and thus sufficient extractable DNA from a single sample or specimen is required for at least long read data generation.	Because of current processing technical limitations, the minimum sample size is about “fly sized” (Drosophila), or 100,000 nucleated cells.
Method of preservation	Good genomes cannot be generated from poor specimens, and BGE/ERGA thus considers specimens as feasible that have been collected	The samples must be snap frozen from live tissue in liquid nitrogen or on dry ice.

	and preserved in a manner that assures excellent maintenance of DNA and RNA (and nuclear) integrity. At present, snap frozen or samples frozen on dry ice or ethanol/dry ice slurry are superior in quality than samples that have been lyophilized, air dried or preserved in solution such as QIAGEN Allprotect, RNALater, RLT Buffer, DMSO, or DESS.	
Sample preserved suitable for Hi-C	Generation of Hi-C long range data requires preservation of intact nuclei and thus samples for Hi-C must use compatible storage methods.	The same feasibility assessment as for “Method of preservation” applies.
Time elapsed from death to preservation	Some organisms may be held alive in captivity/culture for a period of time. This is not what is meant here, but rather the time that has passed since the sacrifice of the specimen to final preservation. The shorter the time span, the better.	The specimen should be sacrificed directly by snap freezing or sacrificed and snap frozen immediately (meaning <5 mins).
Sample preserved suitable for transcriptome sequencing	Samples must be preserved in a way suitable for transcriptomics (Illumina RNAseq or Iso-seq).	The same feasibility assessment as for “Method of preservation” applies.
Storage of sample	The storage of samples between preservation and extraction is essential for high quality DNA, RNA and nuclear work. BGE/ERGA considers only samples that have a validated cold chain of storage since preservation as feasible.	The samples must have been maintained in a validated lower than -70°C cold chain throughout.

Development of different prioritization models

The Sampling and Sample Processing (SSP) committee developed models on how the species prioritization process at stage 2 could be conducted. Essentially a prioritization process is ranking data entries from the most preferred to the least preferred ones. The decision tree principle was chosen as the basis for the first models. In this case, the species are ranked by going through the levels of the decision tree. They are first ranked based on the weights of the category of the first level, then within each of the weights of the first level based on the weights on the second level and so forth. By summing the scores of multiple categories, it also possible to assess multiple categories at the same level together instead of just one. To determine the decision level of each category, the SSP committee conducted a poll among the members participating the discussions on the species selection process (Supplementary Figure 1 and Supplementary Table 2). In the poll, the participants had to answer which category of the six categories they would place at the 1st, 2nd, 3rd, and bottom level of the decision tree. By far the most members of the SSP committee (10 out of 15) ranked the category “Taxonomic representation” at the highest level. It was also the highest ranked category considering the sum of rankings at the first three levels, either as plain counts or as an aggregated sum (Supplementary Table 2). Additionally, no one put it on the bottom level. The categories “Certainty” and “Country representation” were ranked next, but there was no strong difference between the two. The category “JEDI” was ranked by four on the 3rd level and by two at the bottom. The category “Novel leader” was placed on the 3rd level by one and

two at the bottom. The category “Applicability” was ranked at the top level by two, but also by nine at the bottom. Finally, the members also answered if only one category should be allowed per level or multiple categories per level. For this latter question, 40% were for only one and 53.3% for multiple.

Given the results of the poll, the SSP decided to develop several models to reflect the uncertainties of the poll. In total, seven models with a decision tree were developed (Supplementary Table 3). The top level in all models was the category “Taxonomic representation”. At the next two levels, the categories “Certainty” and “Country representation” followed in different orders or in combination. At the next level followed the category “JEDI” as it had the best ratio of top ranked positions to the bottom position in the poll. At the bottom levels, the categories “Novel leader” and “Applicability” occurred in different orders or in combinations.

Supplementary Table 2: Results at which level a category was chosen in the poll among the SSP committee members participating the discussions on the species selection process. Number of times suggested at 1st, 2nd and 3rd and bottom position. Sum = sum of occurrences at first three positions; Aggregate = Weighted sum (1st = 3, 2nd = 2, 3rd = 1) of first three position; Top to bottom = Ratio of aggregated sum of top to number of occurrences at bottom.

	Top					Bottom	Top to
	**1st**	**2nd**	**3rd**	**Sum**	**Aggregate**		**Bottom**
**Certainty**	1	5	5	11	18	1	18.0
**Taxonomic representation**	10	5	0	15	40	0	N/A
**Country representation**	2	5	5	12	21	1	21.0
**Novel leader**	0	0	1	1	1	2	0.5
**JEDI**	0	0	4	4	4	2	2.0
**Applicability**	2	0	0	2	6	9	0.7

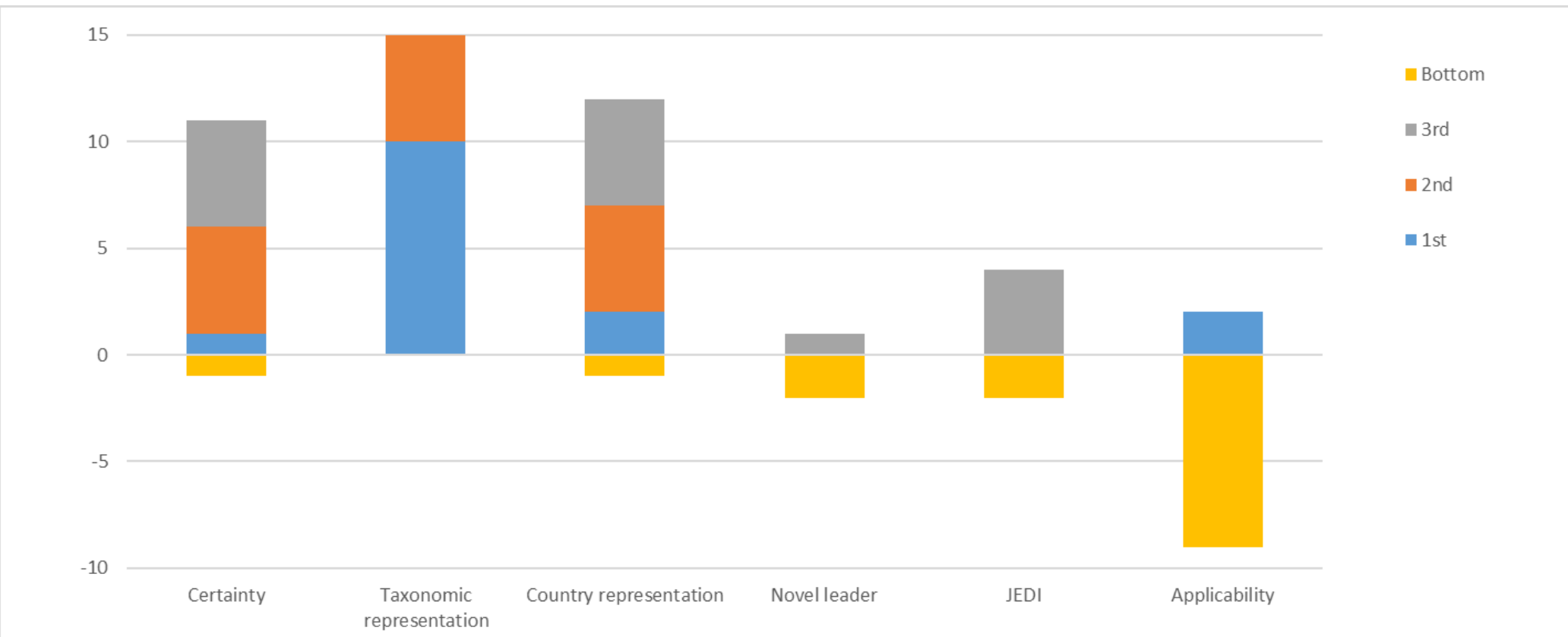

Supplementary Figure 1: Cumulative histogram at which level a category was chosen in the poll among the SSP committee members participating the discussions on the species selection process.

Supplementary Table 3: The different models using a decision tree with one or more categories at each level.

One category per decision level:

**Level**	**Model 1**	**Model 2**	**Model 3**	**Model 4**
1st	Taxonomic representation	Taxonomic representation	Taxonomic representation	Taxonomic representation
2nd	Country representation	Certainty	Country representation	Certainty
3rd	Certainty	Country representation	Certainty	Country representation
4th	JEDI	JEDI	JEDI	JEDI
5th	Novel leader	Applicability	Applicability	Novel leader
6th	Applicability	Novel leader	Novel leader	Applicability

Up to two categories per decision level:

**Level**	**Model 5**	**Model 6**	**Model 7**
1st	Taxonomic representation	Taxonomic representation	Taxonomic representation
2nd	Country representation & Certainty	Country representation & Certainty	Country representation & Certainty
3rd	JEDI	JEDI	JEDI
4th	Novel leader	Applicability	Applicability & Novel leader
5th	Applicability	Novel leader	

Finally, another model was developed, which is a special case of the decision tree. In this case, all categories are treated equally, and the weights are just summed up for each suggested species. It essentially means that all six categories are assessed at one level. One should note that an implicit weighing is present due to the different weights (especially maximum weights) for each category. The maximum score possible across all six categories is 25, the absolute minimum is 4. The suggested species are then ranked from the highest sum to the lowest. This model is called model #8.

Based on the results of the simulated and empirical data (see the next two sections), the SSP committee decided on two models to suggest to the ERGA council for a general decision on which model should be implemented in the species prioritization process of stage 2. These are the models #7 and #8.

Both models resulted in an uneven distribution concerning countries and individuals, with a few countries and individuals being especially favoured. Accordingly, an additional option was suggested. After the prioritization by the model, the top 10 selected species are selected as is, then the best-ranked species of each country, which does not have a species yet considered, is selected followed by the selection of the best-ranked species of everyone suggesting a species and not having been selected yet. The remaining species are ranked in accordance with the model again.

Weighing scheme of each category

At the ranking stage, we grouped different criteria into categories. In the following, we will describe these categories, the included criteria, and the weighting scheme applied. Note, for each category, a weighting scheme has been assigned for different possibilities of combinations of the included criteria. The logical mathematical codes used in the formal description of the weighing scheme of each category have the following meaning: ! = NOT (opposite), || = OR, && = AND. Moreover, the numbers in the weighting scheme refer to the numbers after the criterion in Table 1 in the manuscript and Supplementary Table 1.

Weighing scheme of category "Taxonomic representation"

1 = genus level

2 = family level

3 = order level

4 = class level

5 = phylum level

A weight of 1 is given if a reference genome is already available for the same genus. A weight of 2 if it is available for the same family, of 3 for order, of 4 for class, and of 5 for phylum.

Weighing scheme of category "Certainty"

1 = ! (type locality || close to) && high taxonomic problems && insufficient procedure of identification

2 = ((type locality || close to) || ! (type locality || close to)) && (high taxonomic problems || insufficient procedure of identification)

5 = ! (type locality || close to) && ! (high taxonomic problems && insufficient procedure of identification)

6 = (type locality || close to) && ! (high taxonomic problems && insufficient procedure of identification)

A weight of 1 is assigned if the sample is NOT obtained at OR close to the type locality AND the species is known to have high taxonomic problems AND the procedure to identify the species is insufficient. A weight of 2 is given if the sample is either obtained at OR close to the type locality OR the sample is NOT at OR close to the type locality AND the species is either known to have high taxonomic problems OR insufficiently identified. A weight of 5 is given if the sample is NOT obtained at OR close to the type locality AND the species does NOT have high taxonomic problems AND is NOT insufficiently identified. Finally, the highest weight of 6 is given if the sample is obtained at OR close to the type locality AND the species does NOT have high taxonomic problems AND is NOT insufficiently identified.

Weighing scheme of category "Country representation"

1 = ! (part of the list in the criterion "Countries with fewer genomic resources") && more than one species per country suggested

4 = part of the list in the criterion “Countries with fewer genomic resources” && more than one species per country

5 = one per country

In this category, two criteria assessing the representation across European countries have been combined. One contributes to the knowledge transfer and hence, has been linked to the country of the sample coordinator. The other contributes to an equal representation of species from across Europe and accordingly is connected to the collection site. This means one species can represent two countries in this category.

A weight of 1 is given if the sample coordinator is NOT located in a country listed in the criterion “Countries with fewer genomic resources” (i.e., the list of EU Widening countries) AND more than one species has been suggested for the country of the sample site. A weight of 4 is given if the sample coordinator is located in a country listed in the criterion “Countries with fewer genomic resources” AND more than one species has been suggested for the country of the sample site. A weight of 5 is given if only one species has been suggested for the country of the sample site, and at least one representative of the country is a member of the genome team.

Weighing scheme of category “JEDI”

1 = ! [(7a || 7b || 7c) && (8a || 8b) && (9a || 9b || 9c || 9d || 9e)]

2 = one of [(7a || 7b || 7c) || (8a || 8b) || (9a || 9b || 9c || 9d || 9e)]

3 = two of [(7a || 7b || 7c) || (8a || 8b) || (9a || 9b || 9c || 9d || 9e)]

4 = [7a && 8a && 9a]

5 = [(7b || 7c) && 8b && (9b || 9c || 9d || 9e)]

A weight of 1 is given if the sample coordinator is NOT a woman (7c) OR a nonbinary/trans person (7b) OR prefers not to say (7a) AND does NOT identify as an underrepresented minority (8b) OR prefers not to say (8a) AND the genome team does NOT include representatives of non-scientific interest groups or organisations (9b), OR of Indigenous Peoples (9c), OR persons with disability/handicap (9d), OR citizen scientists (9e) OR does NOT have support from non-scientific interest groups or organisations (9a). A weight of 2 is given if one of the three criteria “Gender of researcher or gender balance of team”, “Researcher of underrepresented minority”, OR “Diversity & inclusiveness of team” is fulfilled; for example, when the sample coordinator is a woman (7c). A weight of 3 is given if two of the three criteria “Gender of researcher or gender balance of team”, “Researcher of underrepresented minority” OR “Diversity & inclusiveness of team” is fulfilled; for example, when the sample coordinator is a woman (7c) and the genome team has support from non-scientific interest groups or organisations (9a). A weight of 4 is given if the sample coordinator does not prefer to say their gender (7a) AND does not prefer to say if they are an underrepresented minority (8a) AND if the team has support from non-scientific interest groups or organisations (9a). A weight of 5 is given if the sample coordinator is a woman (7c) OR a nonbinary/trans person (7b) AND does identify as an equity-deserving group (8b) AND the genome team includes representatives of non-scientific interest groups or organisations (9b), OR of Indigenous Peoples (9c), OR disabled/handicapped persons (9d), OR citizen scientists (9e).

Weighing scheme of category "Novel leader"

0 = ! Novel

2 = Novel

A weight of 0 is given to this category if the sample coordinator has already gotten a species sequenced via BGE/ERGA resources (NOT a new sample coordinator). A weight of 2 is given if the sample coordinator has not already gotten a species sequenced via BGE/ERGA resources (a new sample coordinator).

Weighing scheme of category "Applicability"

0 = ! (< 1 year && > 2 stakeholders)

1 = (< 1 year || > 2 stakeholders)

2 = (< 1 year && > 2 stakeholders)

A weight of 0 is given to this category if the applicability of the genome is NOT less than a year AND NOT more than two stakeholders have an interest in the genome. A weight of 1 is given if either the applicability of the genome is less than a year OR more than two stakeholders have an interest in the genome. A weight of 2 is given if the applicability of the genome is less than a year AND more than two stakeholders have an interest in the genome.

Simulation studies

To test the effect of the different models on the species prioritization, the SSP committee conducted simulation studies. First, three different probability distributions for the weighing scheme of each category were implemented (Supplementary Figure 2). These probability distributions were used to randomly assign a weight for each category and simulated species. In the first probability distribution all weights were drawn with equally probability. In the second one, it was more likely that low weights were assigned and, in the third one, higher weights. Then, all possible combinations of these probability distributions were generated. This resulted in 729 (3^6) combinations. For each combination, 100 datasets with 1,000 species were

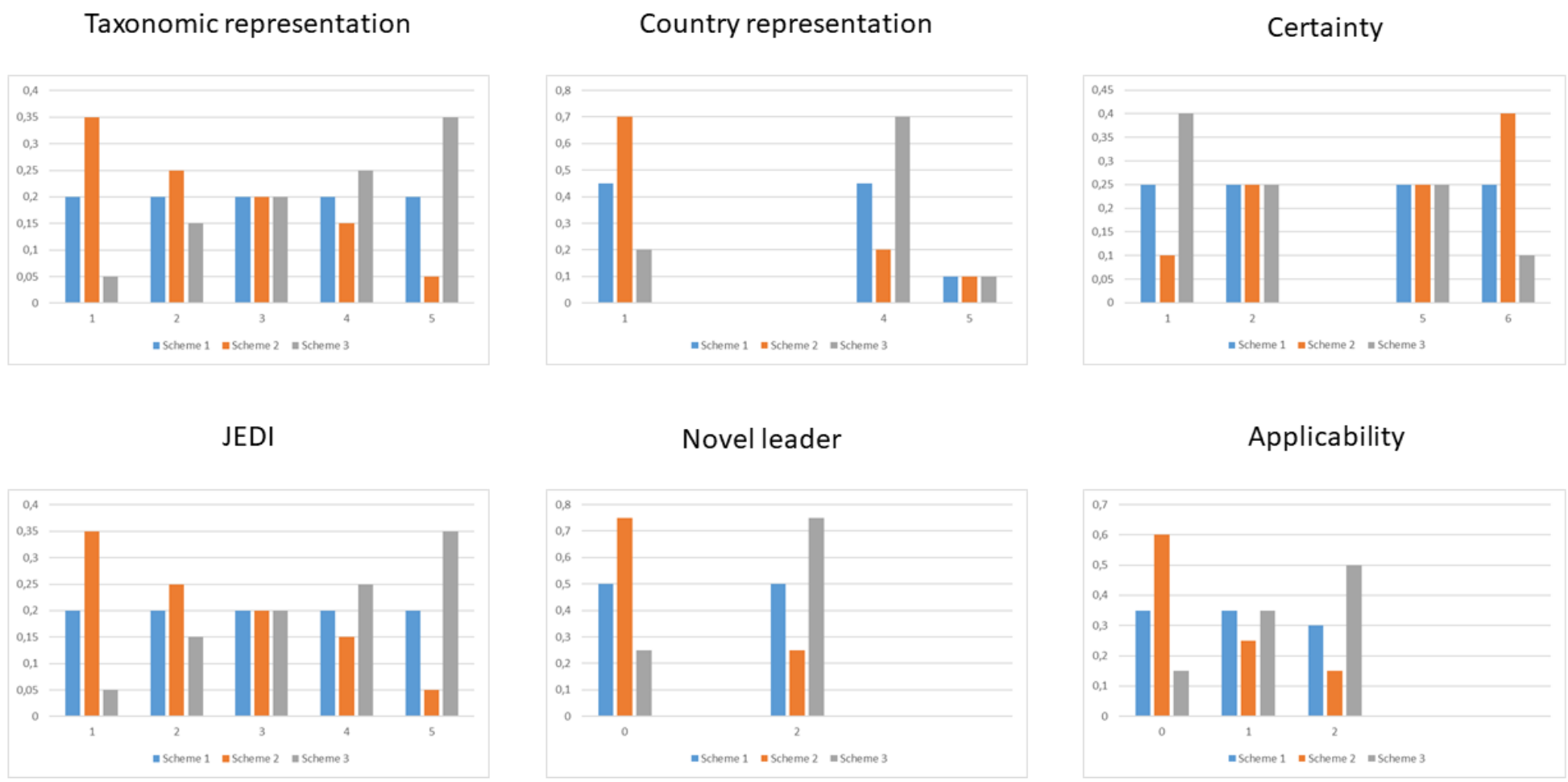

Supplementary Figure 2: The three probability distributions for each category.

generated by randomly assigning a weight for each category to a species given the applied probability distribution. This resulted in 72,900 simulated data sets. The eight models above were applied to each of these datasets and the top 100, 200 and 300 species selected given the ranking of each model.

For each dataset, model, and top-selected species, the enrichment factor for each category was calculated. The enrichment factor measures the relative enrichment of a category in the top-selected species in relation to all species using the following formula:

$$\frac{\overline{Weight_{Top}} - \overline{Weight_{All}}}{Max_{poss.} - \overline{Weight_{All}}}$$

With $\overline{Weight_{Top}}$ being the mean of the weight values of the top-selected species, $\overline{Weight_{All}}$ being the mean of the weight values of all 1,000 species, and $Max_{poss.}$ being the maximally possible value for the category. Hence, it is relative measurement of the maximally possible positive enrichment of a category in the dataset. Negative values are possible and indicate that the category is represented less strongly in the top-selected species. Independent of the dataset, the number of top-selected species and the category, positive values always have an upper limit of 1, while there is no equivalent general lower limit. To explore the 218,700 (3*72,900) results more comparatively, for each combination, model, and number of top-selected species, the mean enrichment factor across the 100 datasets generated for each combination of probability distributions was calculated. For each model and number of top-selected species, heatmaps combined with a hierarchical clustering were estimated for the combinations versus the categories as well as violin plots for each category. Additionally, for each probability distribution of each category, violin plots of each category were generated. This means results were compiled across all the 243 combinations in which any probability distribution could have been present. Next, the mean across the 729 combinations for each model and number of top-selected species for each category were calculated. Of these, a heatmap combined with a hierarchical clustering was generated for the models and number of top-selected species versus the categories.

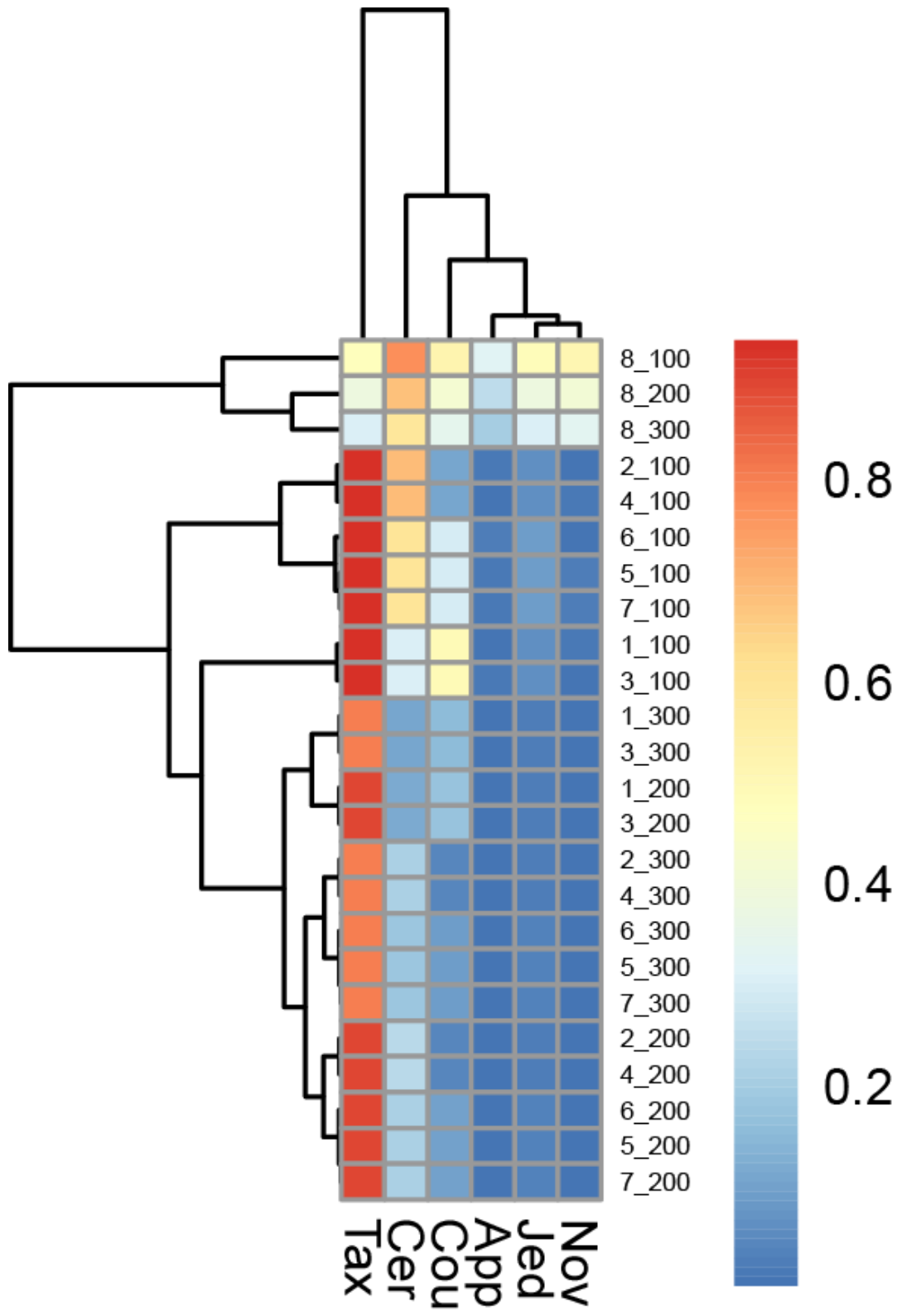

Supplementary Figure 3: Heatmap of simulation studies of the enrichment of the different categories given the models (first number, 1-8) and number of selected species (second number, 100, 200 or 300). App = Applicability, Cer = Certainty, Cou = Country representation, Jed = JEDI, Nov = Novel leader, Tax = Taxon representation.

This last heatmap allows exploring the results of the simulation studies on a more general level. First, model #8 is clearly set apart from all other models (Supplementary Figure 3). Moreover, all categories are more or less equally enriched with the strongest enrichment in Certainty and the least in Applicability.

Among the seven other models, the next split is between the selection of only 100 out of 1,000 species (10%). The models #1 and #3 are thereby placed a bit closer to the other numbers of top-selected species than the other five. The major difference between these is that Country representation is more strongly enriched than Certainty, while it is the other way around in the models #2, #4-7. Given the models, this to be expected. Moreover, in the models #5-7, which assess both categories together, Certainty has a stronger influence than Country representation. Also, for 200 and 300 selected species, the models #1 and #3 are set apart from the other five, but less prominently. Generally, a similar pattern occurs as with the 100 selected species. As most likely 50 out of around 250 species will be selected at the 2nd stage in the first round (20%), the results from the 200 selected species are presented in following. Moreover, models #1 and #3, #2 and #4, as well as #5-7 have no strong differences, and accordingly the discussion is restricted to #1, #2, #7 and #8.

Assessing the enrichment of the four models shows that for models #1, #2 and #7 Taxon representation is most strongly enriched with a median value close to 1 (Supplementary Figure 4). However, for a few combinations of probability distributions it goes down to about 0.75. The last three categories are JEDI, Novel leader and Applicability with median values close to 0 and narrow distribution around it. Even though the heatmap analyses differentiated between the models with Country representation and Certainty is not very strong as the median values are only slightly higher for the one than for the other. The differences are in the skewed distributions towards higher values. Certainty has a stronger enrichment in a few combinations of probability distributions even in the model #1, but in contrast to the models #2 and #7 are these only outliers (see the boxplots). On the other hand, the distribution is it more narrow for Country representation in the models #2 and #7. Concerning these three models, the differences are minor and model #7 seems to be the best compromise between the models #1 and #2 given Country representation. The median values of Country representation and Certainty are very similar as well as the distributions.

Not surprisingly, the picture looks completely different for model #8. For the categories Country representation, JEDI, Novel leader and Taxon representation, the median values of around

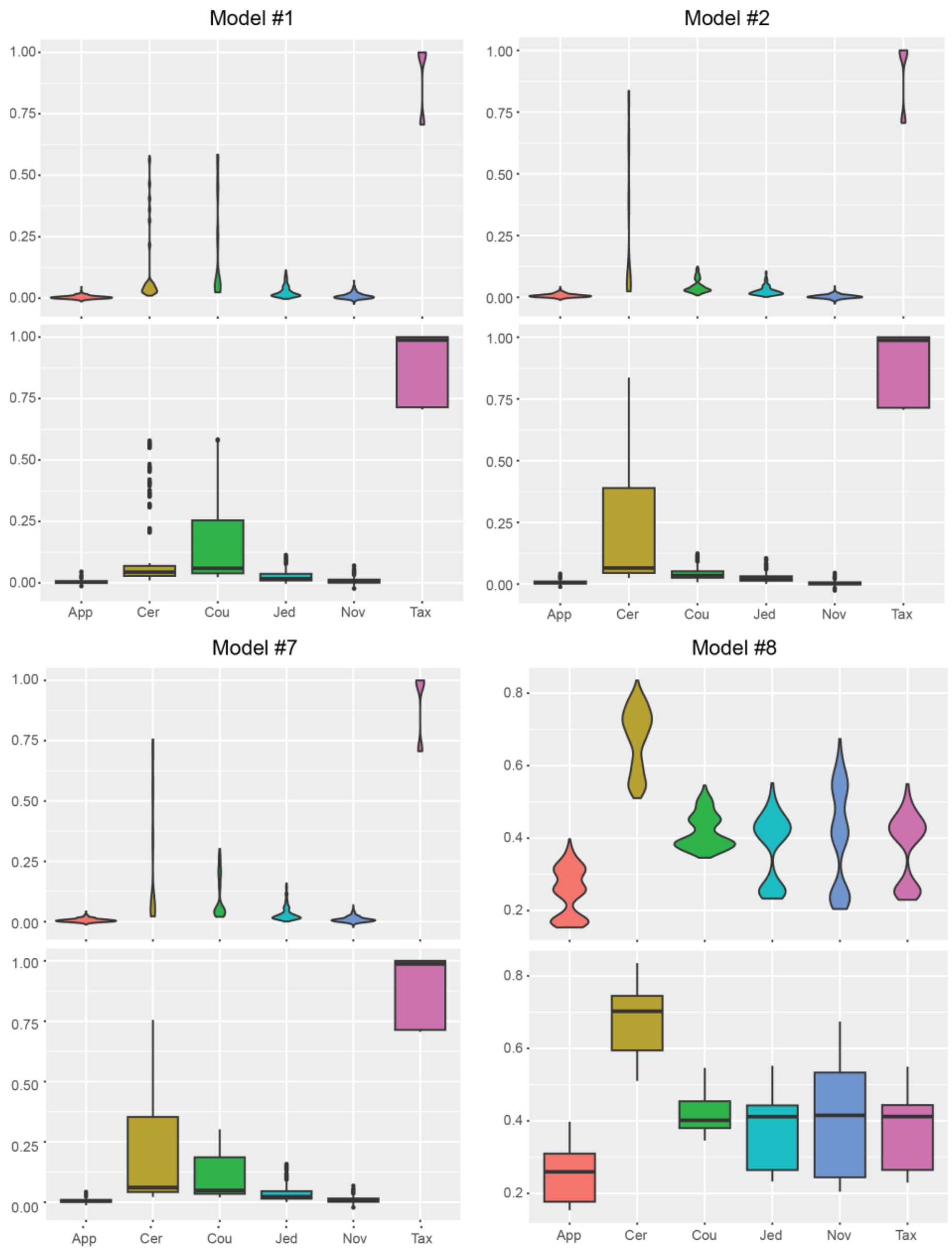

Supplementary Figure 4: Violin and box plots of the enrichment for the different models and 200 selected species. Abbreviations see Supplementary Figure 3.

0.4 and distributions are relatively identical. The distribution of Country representation is narrower and of Novel leader broader. Applicability is clearer lower in both the median values and its distribution, while it is the opposite for Certainty. The latter is clearly favored with a median enrichment of 0.7 (70%). Hence, given the simulation studies model #7 and model #8 would be good candidates to choose among.

Empirical data

In total, 387 species were suggested. After excluding genomes for which a reference genome or with ongoing genome projects, 319 species remain. Excluding all the species for which no voucher could be provided resulted in 236 suggested species and accordingly 151 species were excluded at stage 1. Of these, one species was suggested six times and one species

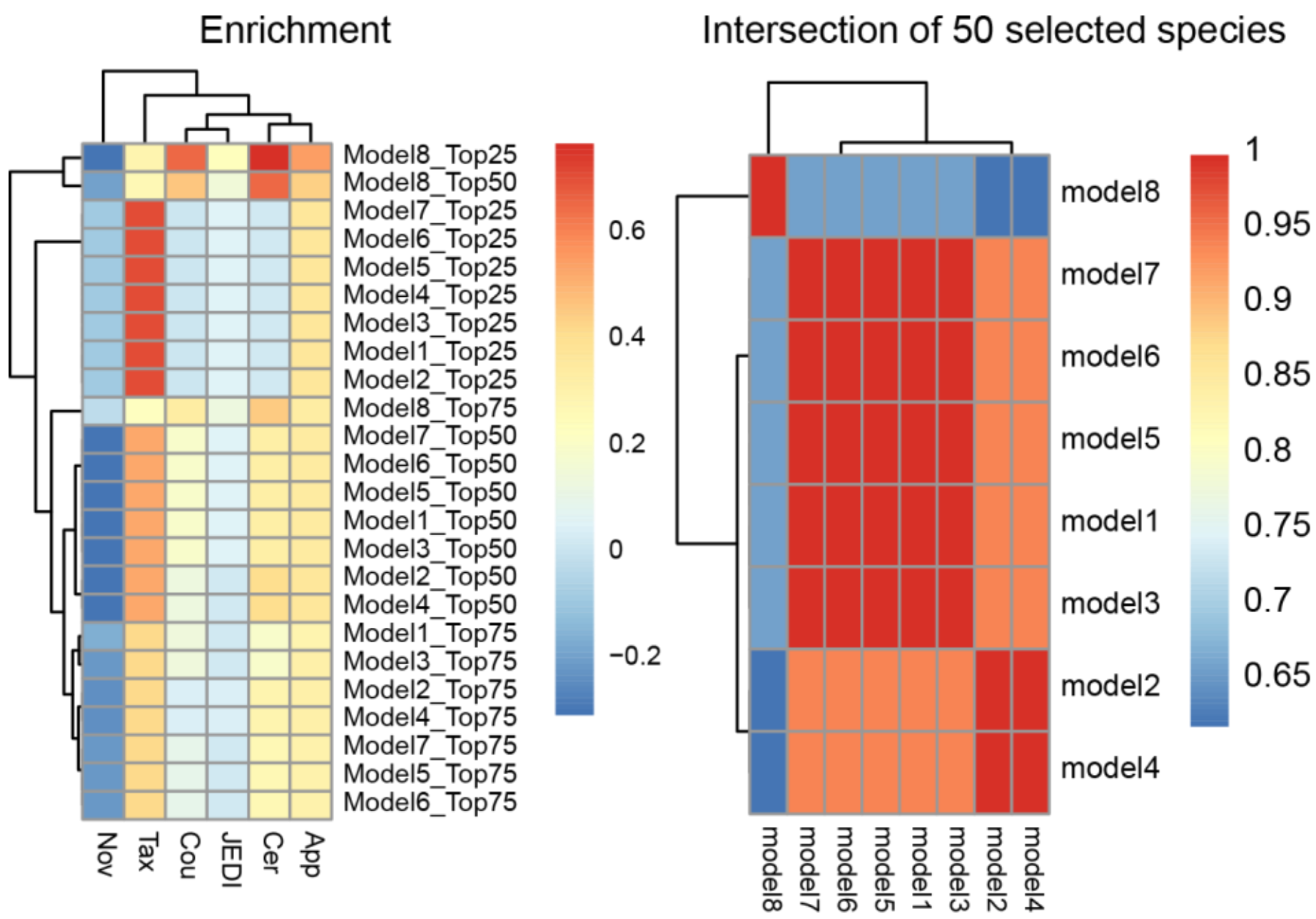

Supplementary Figure 5: Heatmap of the enrichment factor of the different species selection and models versus the categories and heatmap of the intersection between the 50 species selected by the eight different models. Abbreviations see Supplementary Figure 3.

twice. Hence, the actual number of suggested and included species for the prioritization at stage 2 is 230.

After the exclusion stage, the empirical data were generally analyzed similar to the simulated data. We top-selected roughly 10% (25), 20% (50) and 30% (75) of species and used the enrichment factor to assess the outcome for the eight models. Additionally, the intersection of species selected between the models within the cohort of selected species as well as the proportion of the different options of certain criteria before and after the selection were calculated.

Similar to the simulation data, model #8 is clearly different from all other models (Supplementary Figure 5). Within model #8, the differences are more pronounced with fewer species selected. The strongest enrichment has Certainty followed by Country representation and Applicability. While JEDI and Taxon representation have a low enrichment or none, has Novel leader negative values. In the case of the other seven models, the separation is between the number of selected species. Within 25 selected, there is no difference between the models. Taxon representation is strongly enriched and Applicability intermediately, while all others are not enriched. Selecting 50 species, enrichment of Taxon representation is not as strong, while enrichment of Certainty, Country representation and Applicability are increased to intermediate values. JEDI shows no enrichment, while Novel leader has strong negative values. Moreover, models #2 and #4 are slightly different from the other five. Finally, the picture with 75 species looks similar, but for Taxon representation and Novel leader the values are not so strong any longer and the difference of models #2 and #4 is a bit more pronounced.

Looking at the intersections of the 50 selected species shows that the overlap of selected species between the model is actually very high with equal to or more than 62% of species shared among any pair of models (Supplementary Figure 5). Nonetheless, three groups can be recognized. One contains again only model #8. The other one models #2 and #4, which

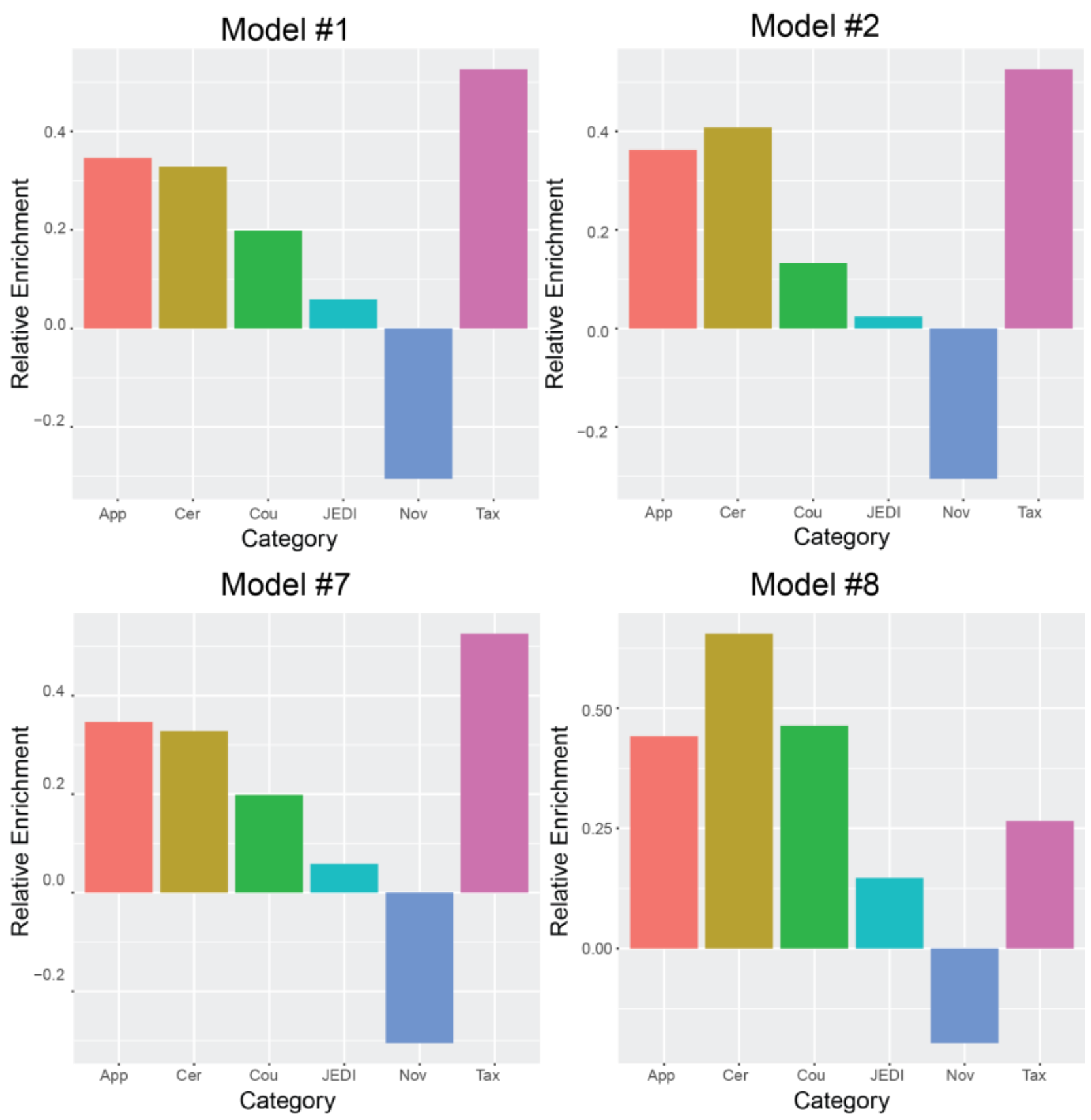

Supplementary Figure 6: Bar charts of the enrichment for the different models and 50 selected species. Abbreviations see Supplementary Figure 3.

select the same species. The last group contains the remaining five ones, which also select the same species. The similarity in species selection between these two last groups is also very high with 94%. Hence, like with the simulation studies the similarity between the models #1-7 is very high.

Like in the simulation studies, the models #1, #2, #7 and #8 for 50 selected species (~20%) are compared. Not surprisingly given the same selected species, the enrichment for models #1 and #7 is identical (Supplementary Figure 6). The strongest enrichment is for Taxon representation with about 0.5. Surprisingly, the second strongest enrichment is Applicability, even though it is at the lowest level in the two models. This is followed by Certainty, and then Country representation, even though the latter is one level higher than the former. As expected, the category Novel leader being the second lowest has negative values and JEDI as the 4th level category has little enrichment. In model #2, the major differences to the other two models are that Certainty has the second highest in accordance with its 2nd level position and that Country representation is even smaller. Applicability remains high.

For model #8, the enrichment is different (Supplementary Figure 6). Certainty has the highest enrichment followed by Applicability and Country representation. Taxon representation is only about half of the other three models, while JEDI is much stronger. Novel leader is still negative, but not as strongly. Overall, in this model enrichment is more evenly distributed than in the other three models similar to the simulation studies.

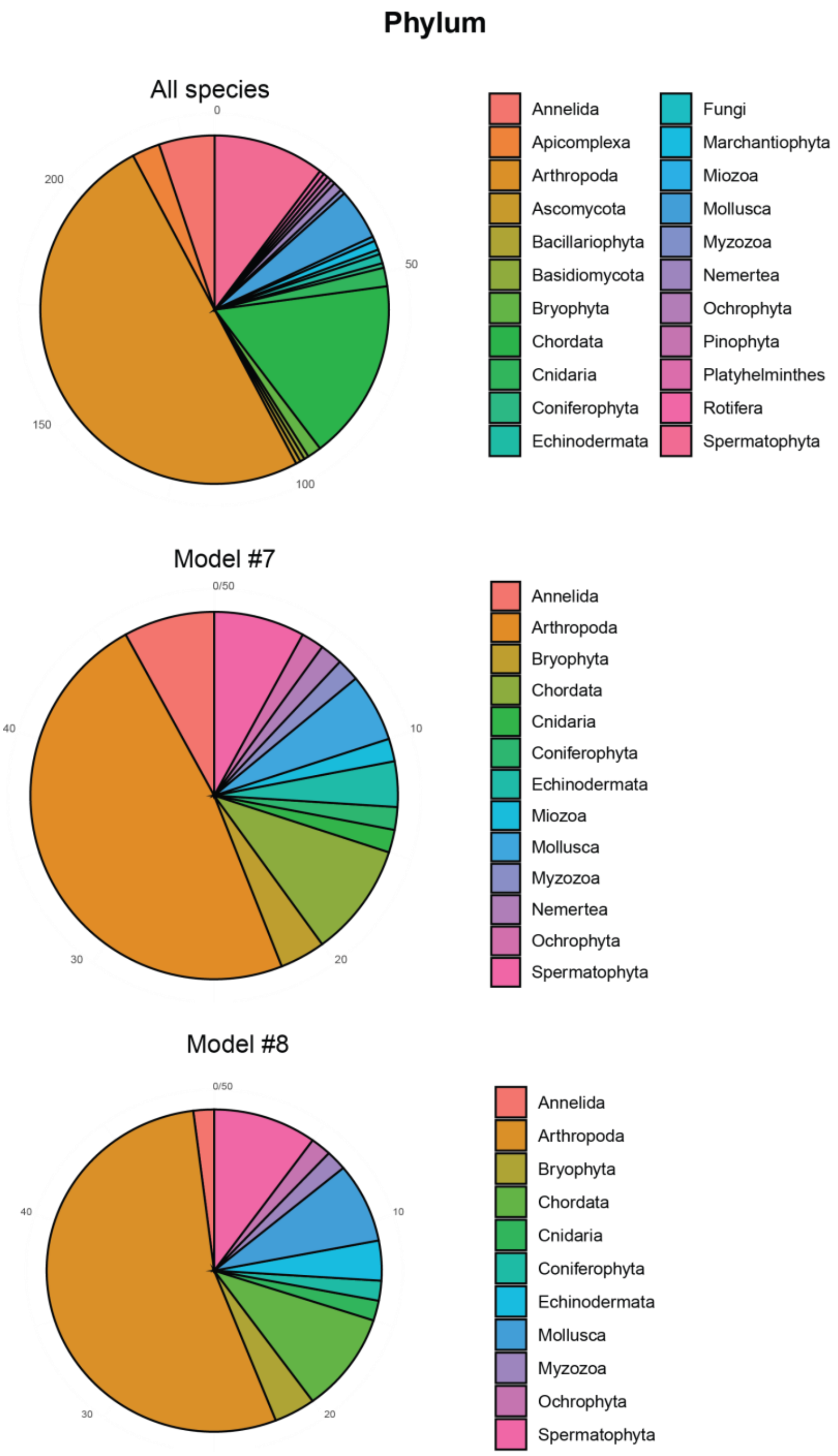

Supplementary Figure 7: Taxon representation at the phylum level for all species and the 50 selected species using models #7 or #8.

In following, we will show the change in proportions for a few criteria by applying models #7 and #8. Looking at the Taxonomic representation by phyla, the number of phyla is substantially reduced with both models (Supplementary Figure 7). Taxa such as Apicomplexa, Ascomycota, Bacillariophyta, Basidiomycota, Marchantiophyta, Pinophyta, Platyhelminthes and Rotifera are not selected. Concerning model #7, this large exclusion is probably because across all species only two had a close reference genome available at the phylum level and 24 at the class level. The remaining 24 were at the order level and accordingly other criteria than Taxonomic representation became more important for these. Using model #8, eight are at the genus level, another nine at the family level, 19 at the order level, 14 at the class level

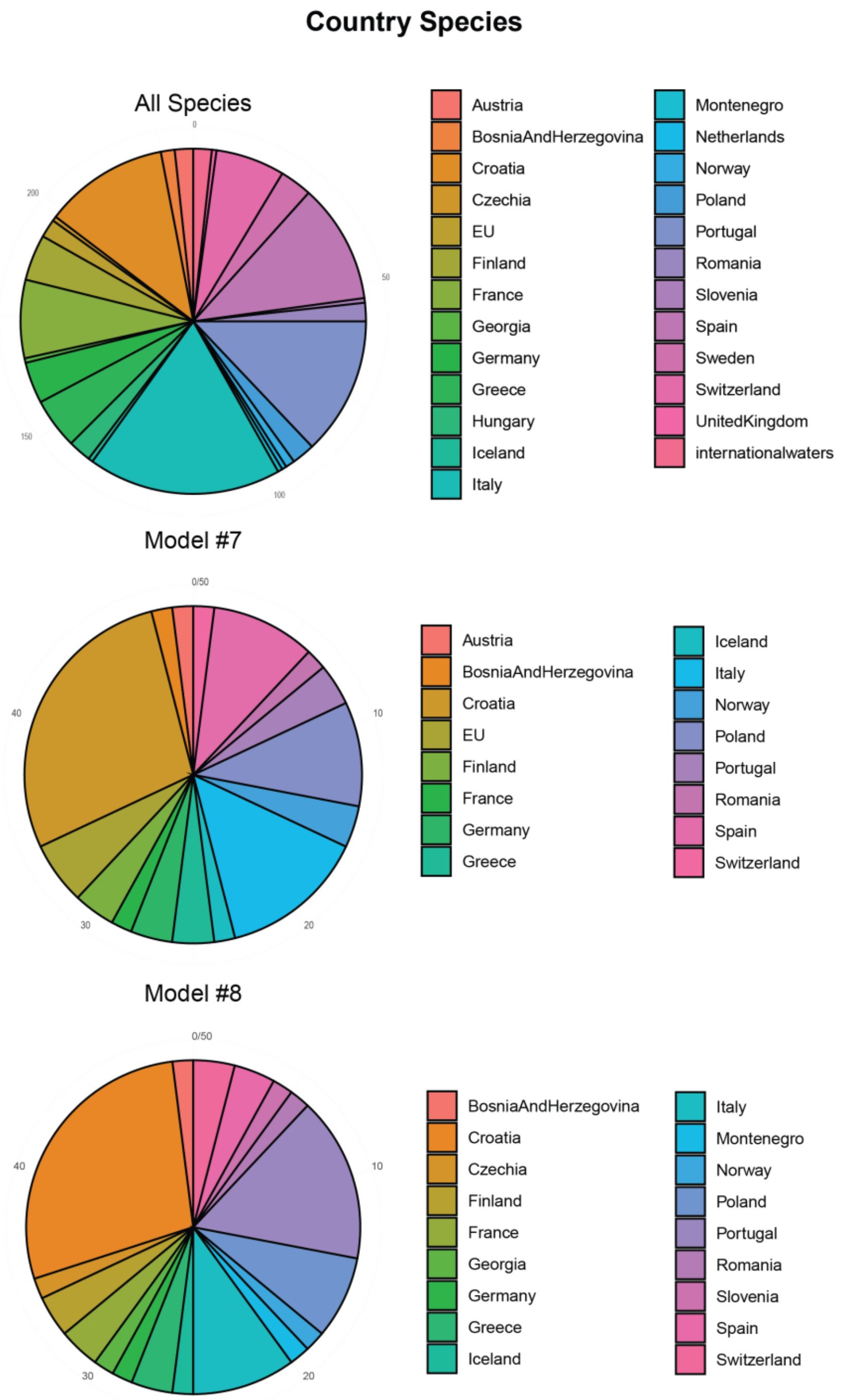

Supplementary Figure 8: Representation of the countries, where the species are collected, for all species and the 50 selected species using models #7 or #8.

and none at the phylum level. This highlights that model #8 clearly selects species differently than the other seven models.

A criterion, which was only indirectly included in the categories, is the country of the collection site. Countries for which there was only one suggested species got a higher score by point when a person from the country is part of the genome team. Six species fulfilled this criterion and with model #7 only one was among the 50 selected. For model #8, it was four species. Of 25 suggested countries (including EU and international waters), model #7 selected 16 countries with the most favored countries being Croatia, Italy, Poland and Spain

(Supplementary Figure 8). Model #8 selected 18 with Croatia, Italy, Poland and Portugal being the strongest represented.

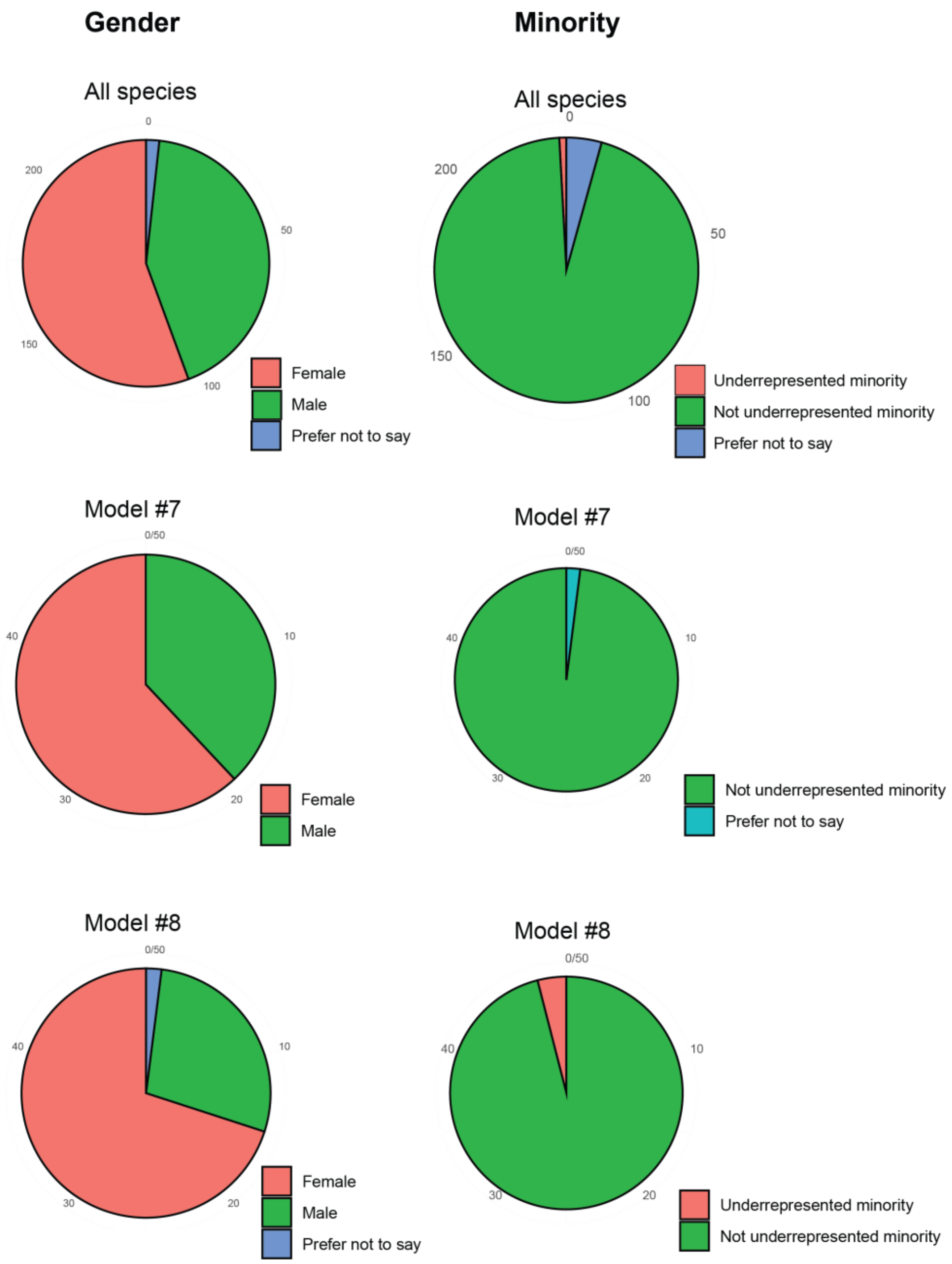

Supplementary Figure 9: Gender and minority representation for all species and the 50 selected species using models #7 or #8.

With respect to gender, both models prioritize female sample coordinators slightly, while model #8 put more weight on underrepresented minorities than model #7 (Supplementary Figure 9). Model #8 also favors slightly more diversity in the genome team than model #7 and the proportion of purely scientific genome teams is smaller than in model #7 (Supplementary Figure 10). Among the top 50, both models have three to four individuals contributing with many species (Supplementary Figure 10).

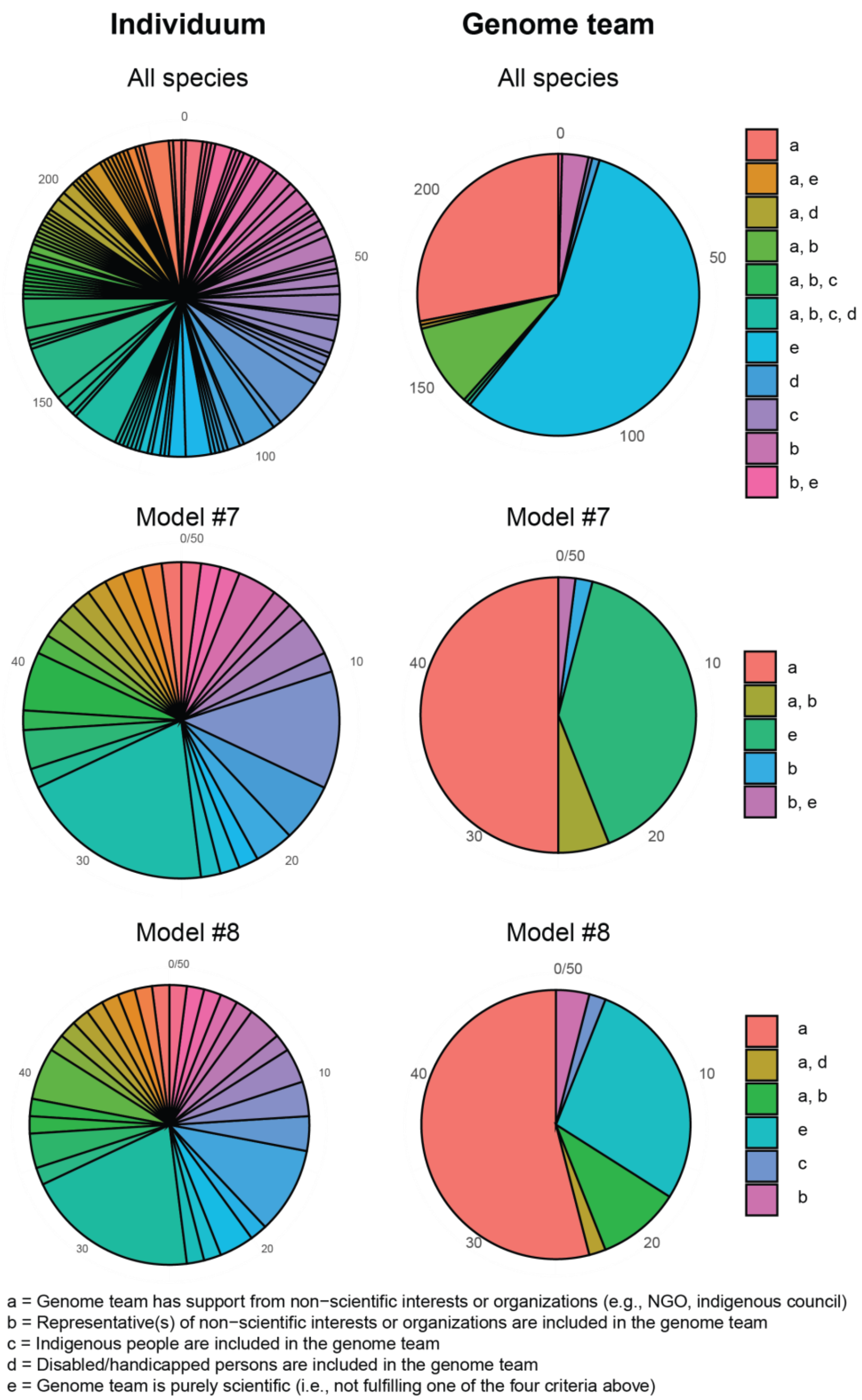

Supplementary Figure 10: Proportion of individual researchers leading teams and diversity of the genome team for all species and the 50 selected species using models #7 or #8.

Concerning the certainty of species identification are there no known taxonomic problem among the 50 selected by model #8, while it is comparable to all species in model #7

(Supplementary Figure 11). Both models enrich species, which are collected close to the type locality (Supplementary Figure 11). Model #8 shows a slightly higher enrichment than model #7. Both models reduce taxonomic identification, which is based on only one procedure except for availability of a key for the region (Supplementary Figure 12). This one is enriched.

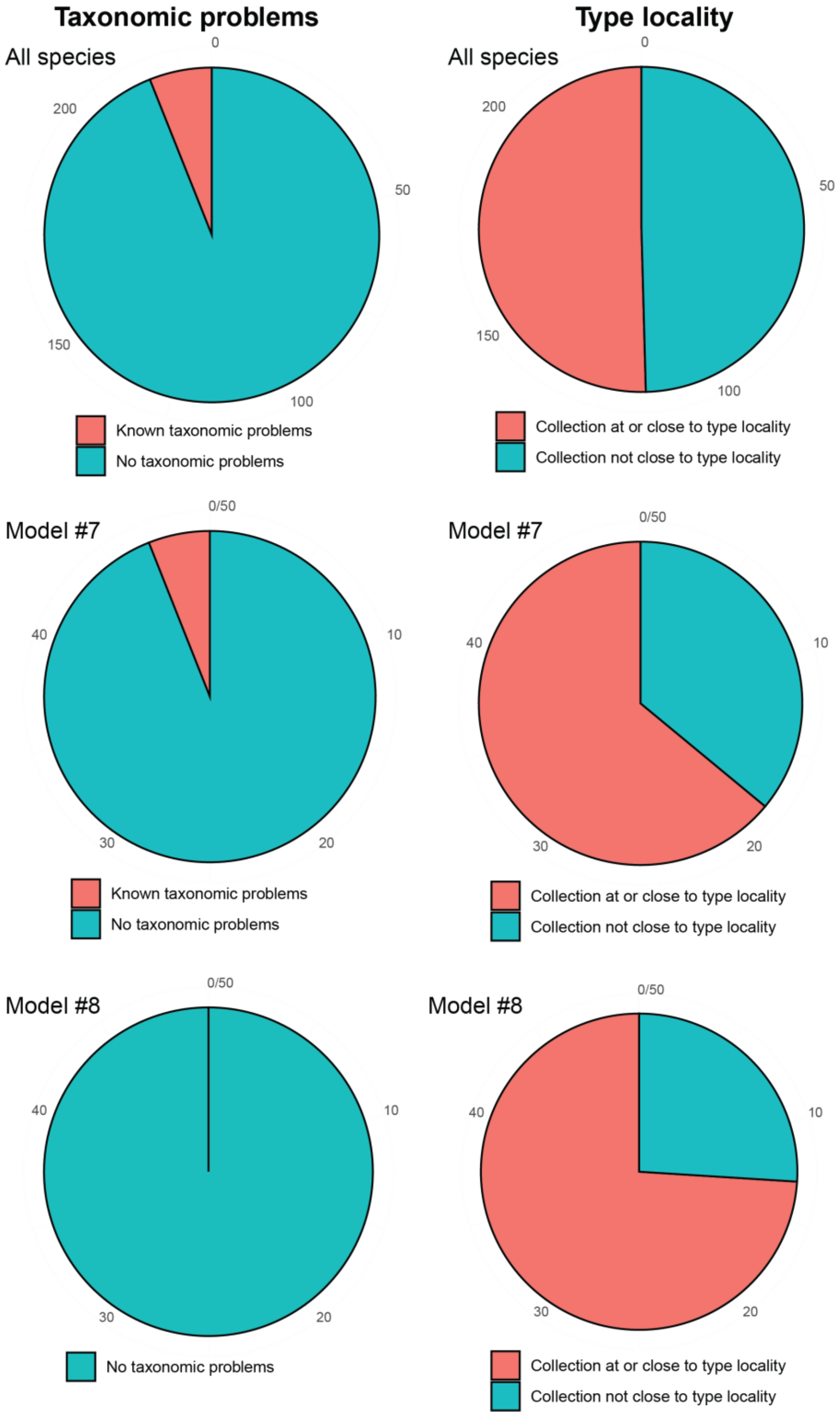

Supplementary Figure 11: Proportion of taxonomic problems and proximity to the type locality for all species and the 50 selected species using models #7 or #8.

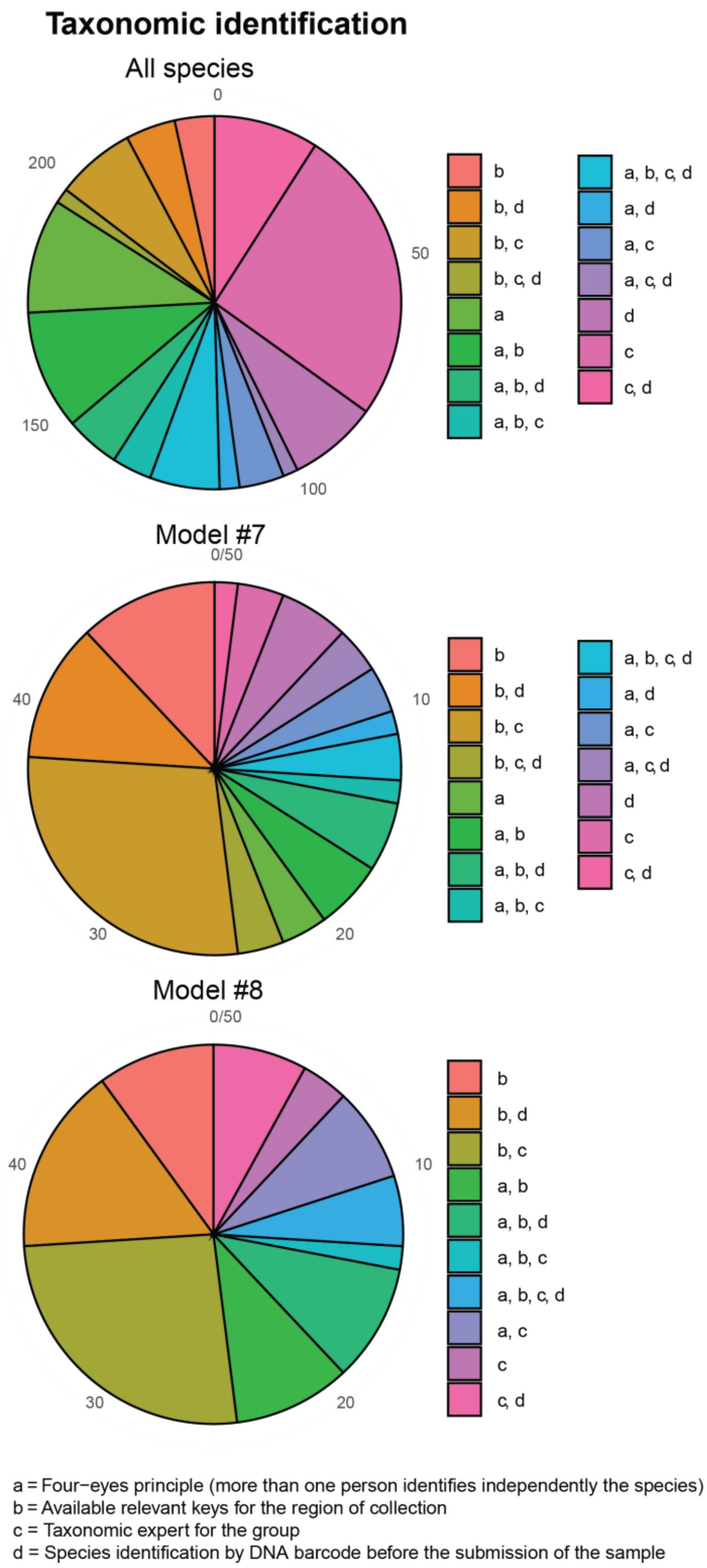

Supplementary Figure 12: Proportion of procedures applied to species identification for all species and the 50 selected species using models #7 or #8.

Additional step for prioritization

Given the uneven distribution concerning countries and individual, we also suggested an amendment to the purely model-based species selection.

1.) Keep the top 10 selected species,
2.) Among the remaining species, move up the best-ranked species for each country which has not been included already among the top-10 species,
3.) Among the remaining species, move up the best-ranked species for each individual researcher suggesting a species, which have not been included among the top-10 species and best-ranked species based on country,
4.) Rank the remaining species in accordance with the model.

The country of collection site is relevant here. Moreover, the ranking within the best-ranked species by country as well as by individual researcher is in accordance with the ranking by the model of the species prioritization process.

Considering all models, this changes the general similarity between the different models slightly. Model #8 is clearly different from the other models considering both the enrichment factors as well as the selected species. However, the intersection of the 50 selected species to the other models increases from at least 62% to 68%. Models #2 and #4 are still slightly different from models #1, #3, #5-7 in both. Additionally models #1 and #3 are slightly different to models #5-7. Within each of these three groups, all selected species are identical. The intersection of group of models #5-7 to the other two is 98%, while the other two groups are 96% identical. As models #5-7 are still intermediate between models #1/#3 and models #2/#4, we still concentrate on models #7 and #8 in the following.

Not surprisingly, the enrichment factors change (Supplementary Figure 13). For model #7, the category Certainty decreases only a little bit, while Taxonomic representation, Applicability, Country representation and JEDI decrease substantially. Novel leader increases strongly but is still negative. For model #8, the factors Taxonomic representation and Applicability decrease only a little. Country representation and JEDI decrease substantially, while Certainty increases strongly. Novel leader increases also but remains negative. Interestingly, in both cases the score of Country representation decreases, even though more countries are selected (Supplementary Figure 14). This is due that the scores for Country representation mostly differ by the score for countries with fewer genomic resources. Accordingly, the count of the score value of 1 increases substantially in both cases (Supplementary Figure 13). Additionally, even though the score of 5 also increases slightly, it cannot counterbalance the increase of scores with 1.

The taxon representation is slightly increased from 13 to 16 and 11 to 14 phyla for both models (Supplementary Figure 14). Moreover, the proportion of Arthropoda is reduced. Not surprisingly, all countries are represented in both models now and the distribution is generally more even across the countries (Supplementary Figure 14). Similar results were obtained for individual researchers (Supplementary Figure 14).

Concerning the Certainty, the number of species with known taxonomic problems is not strongly changed (Supplementary Figure 15). However, in both models the number of species collected at or close to the type locality decreases and more strongly in model #7 (Supplementary Figure 15). Additionally, for the type of species identification, the taxonomic

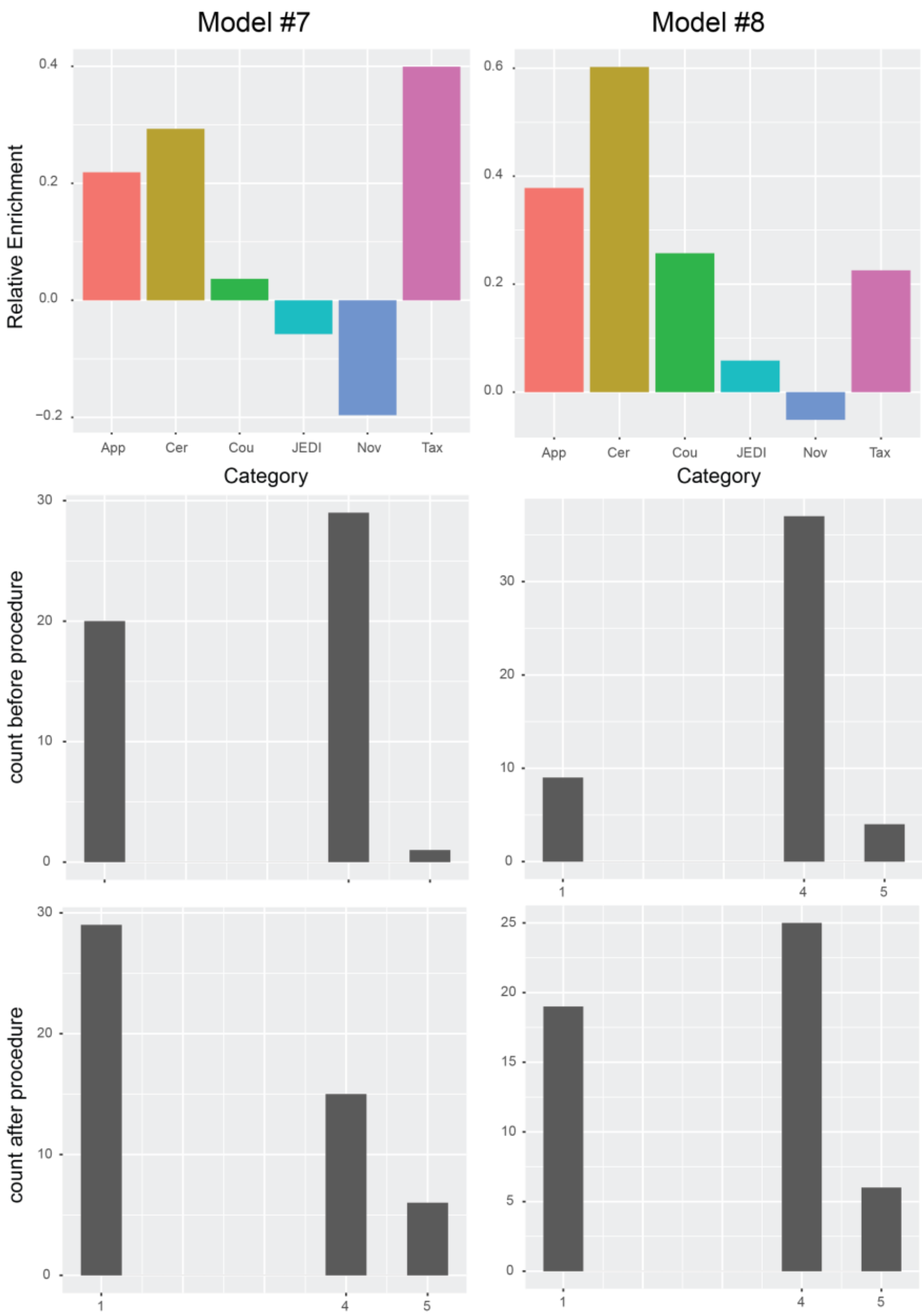

Supplementary Figure 13: Bar charts of the enrichment for the models #7 and #8, 50 selected species and additional prioritization of countries of collection site and individual researchers. Histograms of the category "Country representation" for the two models for and after the additional option. Abbreviations see Supplementary Figure 3.

identification, which is based on only one procedure, is more strongly reduced, especially for availability of a key for the region (Supplementary Figure 15).

With respect to JEDI, the proportion of male researchers strongly increases in both models, especially in model #7 (Supplementary Figure 16). On the other hand, representation of minorities increases slightly in both models (Supplementary Figure 16).

Given all these results, the SSP committee concluded that the models #1-7 are very similar to each other and that of these model #7 captures best the different opinions on priority in the

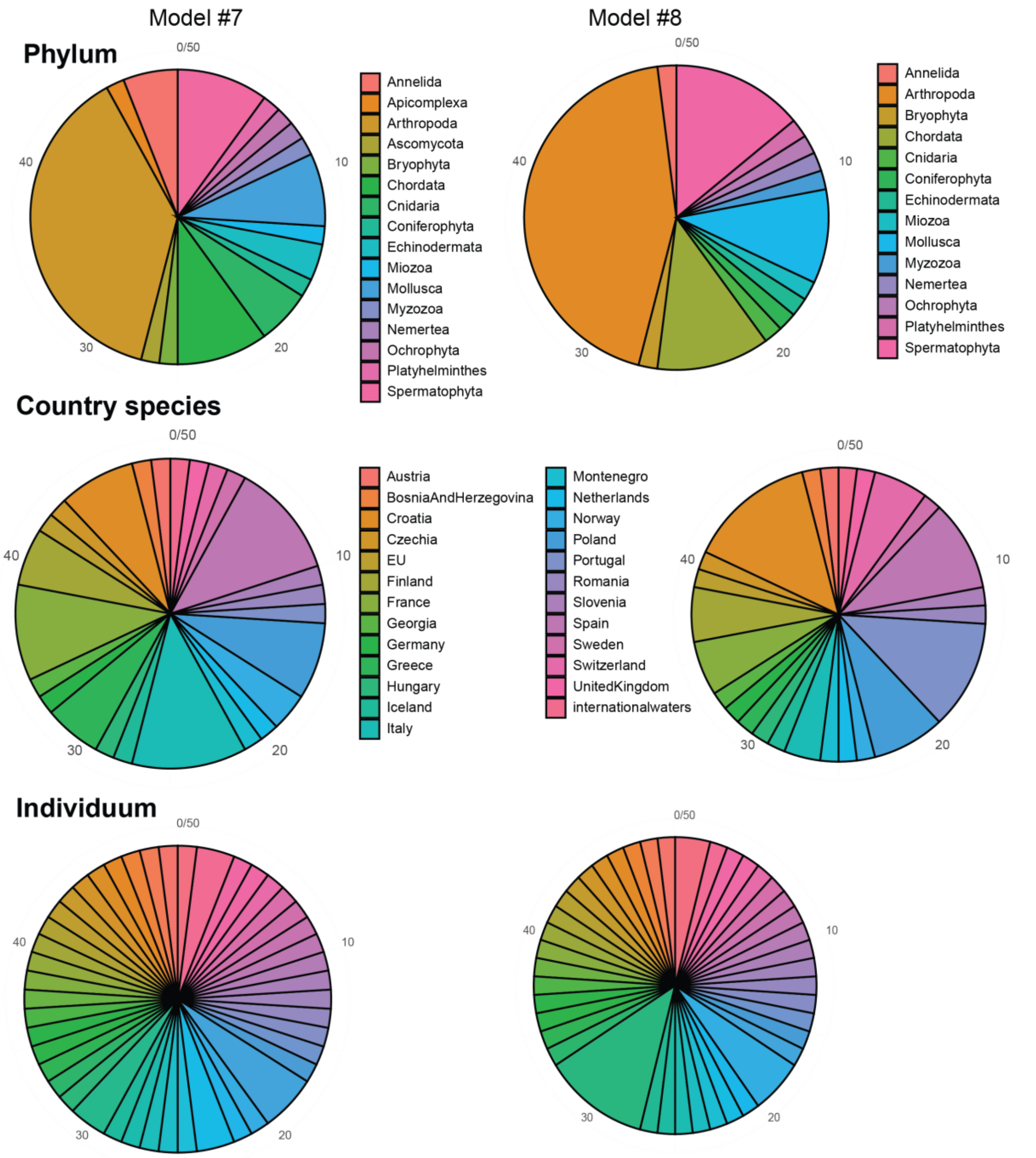

Supplementary Figure 14: Taxon representation at the phylum level, representation of countries of collection sites and individual researchers for models #7 and #8, the 50 selected species after the additional option.

committee. It was therefore suggested that the SSP committee asks the ERGA council to vote if model #7 or #8 shall be used at stage 2 as well as the additional option. Moreover, the council should decide if the additional option should be implemented directly after the model or after the feasibility at stage #3.

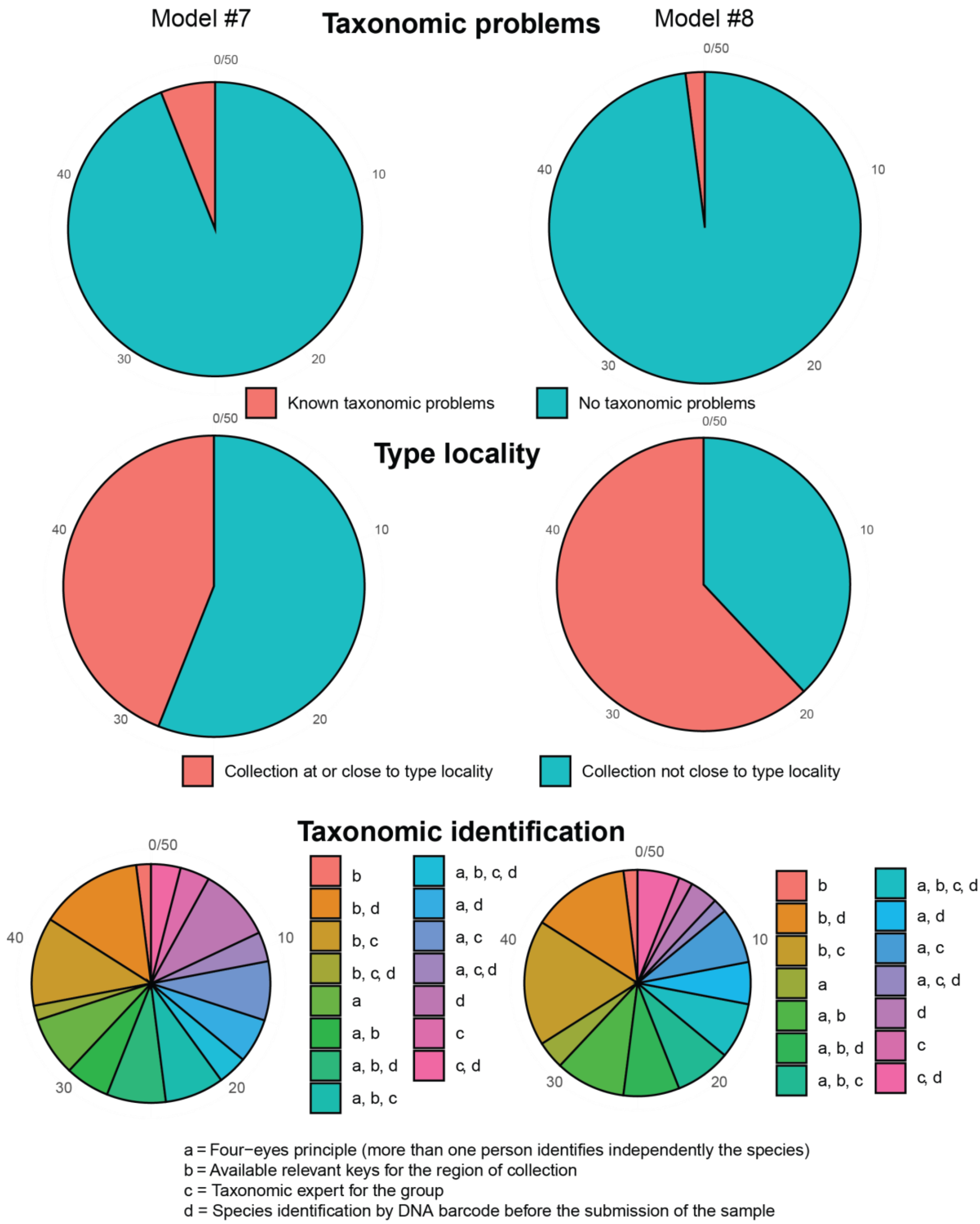

Supplementary Figure 15: Representation of species with taxonomic problems and at or close to the type locality as well as the procedures used for species identification for models #7 and #8, the 50 selected species after the additional option.

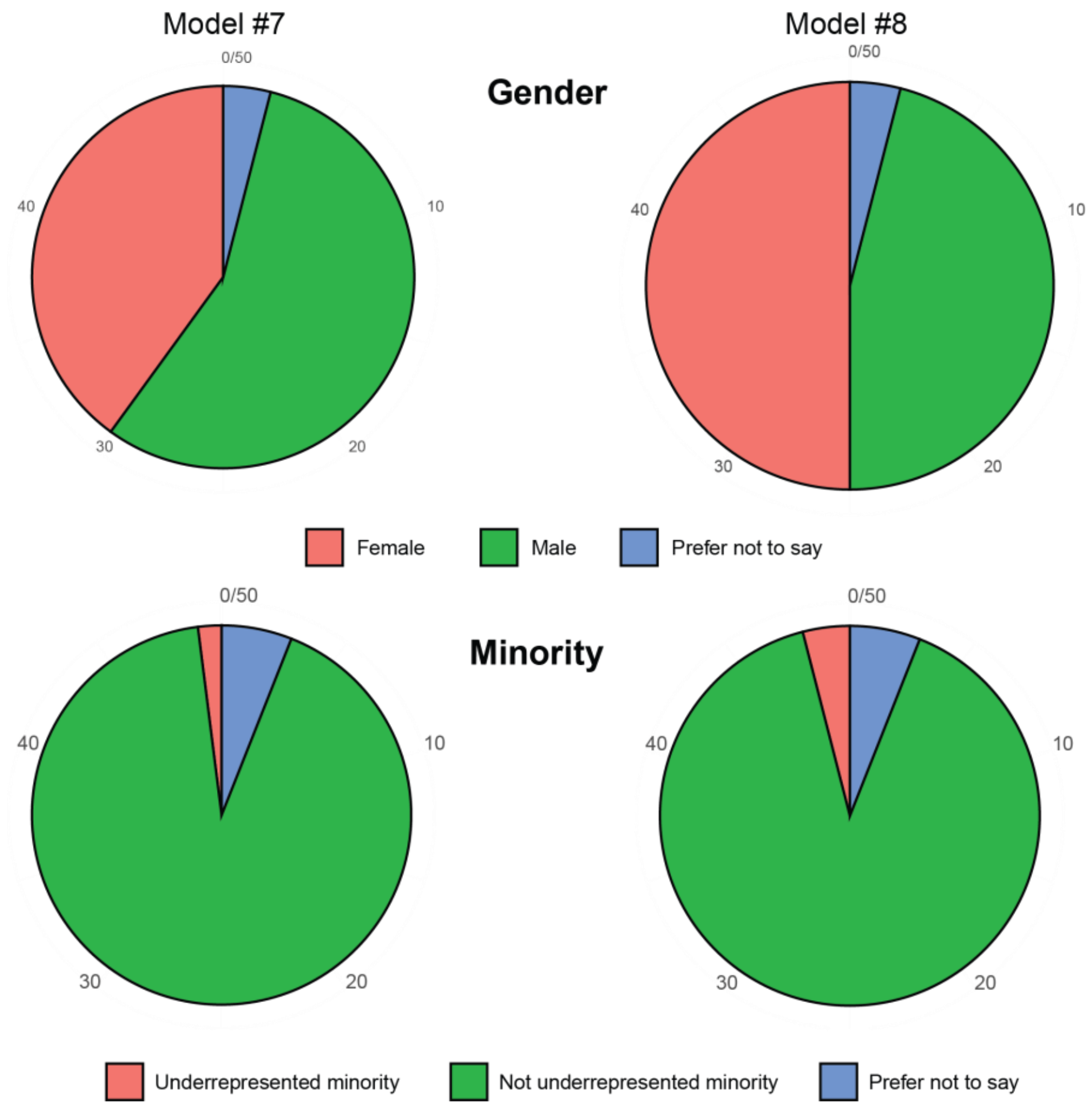

Supplementary Figure 16: Representation of gender and minorities among the selected genome teams for models #7 and #8, the 50 selected species after the additional species prioritization.

Decisions by the ERGA council

Considering the results based on the simulation and empirical data together, the council was asked to vote on model #7 and model #8. Model #8 is different from the other seven in its procedure and the results, while model #7 captured most of the uncertainty within the working group with respect to the placement on the decision trees for these four categories (Supplementary Figure 1 & Supplementary Table 2). Out of the 70 council members, 39 voted, and hence the participation was 55.7%. Model #7 achieved a clear majority of 89.7%, while 7.7% favoured model #8 and 2.6% abstained. Accordingly, model #7, as shown in Figure 1, was implemented.

The decision of including additional steps of ranking was also made by the ERGA council. A clear majority was in favour of implementing the additional steps, with 43.6% favouring it for both countries and individual researchers, 33.3% for only countries, and 2.6% for only individual researchers. Only 5.1% voted against it and 15.4% abstained. A relative majority (44.7%) wanted to implement the additional step after the model-based species ranking of stage 2, but before the feasibility check (stage 3). In contrast, 36.8% wanted it after the feasibility check and 18.4% abstained.

Questionnaire for the nomination in round #2

I have read and understand the ERGA Privacy Policy and agree with the terms and conditions. To proceed with completing this form you will need to select 'Yes' below

Yes No

ERGA privacy policy

Do you consent with your personal data being processed as described in the Consent Statement? To proceed with completing this form you will need to select 'Yes' below

Yes No

Consent Statement

Personal information

Email* (required)

Please provide only the e-mail of one person as the contact person.

First name(s)* (required)

Please provide only the name of one person as the contact person.

Last name(s)* (required)

Please provide only the last name of one person as the contact person.

Affiliation / University / Institute* (required)

Full international shipping address to which the tubes for collecting samples shall be send if the suggested species is selected.* (required)

The country in which the institution is located* (required)

Are you already a signed-up ERGA member?

Yes No

Click here to sign up

Species suggested

Please fill in a separate form for each species you are suggesting. Do not suggest more than one species per genus (within-genus replicates will not be included in the final list). If you are suggesting one species from a genus, but you do not yet know the specific species or within the genus species could be exchangeable, please write "sp." into the field "Species epithet". If you are suggesting one species that is known to be distinct but has not yet been described and given a formal name, please write "spnov." in the field "Species epithet".

NCBI taxonomy identifier for the species. Please obtain this digit code from the Taxonomy Identifier by searching for the species and genus (Latin names) and without the full taxid lineage. For instance, the Eurasian magpie, Pica pica, has the taxonomy ID 34924. If NCBI provides a preferred name, please use that name below. If the species does not have a Taxonomy ID yet, please contact Thomas Marcussen, so that he can advise you how to obtain a Taxonomy ID for your species or genus.

Taxonomy ID for genus* (required)

Genus name* (required)

Taxonomy ID for species* (required)

Species epithet* (required)

Clicking on the Taxonomy ID in the Taxonomy Identifier will show you the full lineage, by hovering over the names in the lineage with the mouse you can see which Linnaean rank it is. Please use these names for Family, Order, Class and Phylum here.

Family* (required)

Order* (required)

Class* (required)

Phylum* (required)

Published reference genomes* (required)

○ Yes ○ No

Is there a published genome available for this species and does it fulfill the EBP minimum standard (i.e., contig N50 > 1 Mb, scaffold N50 > 10 Mb or chromosomal (when chromosomal N50 < 10 Mb) and BUSCO > 90%)?

You can check this on GoaT if you do not know.

There is an Illustrated GoaT Search Tutorial on the GoaT Help page along with instructions on how to use the platform.

Redundancy* (required)

○ Yes ○ No

Is the species already under consideration for the generation of a high-quality genome considering the EBP minimum standard in the near future, e.g., by other consortia such as DToL or EBP-Nor?

Species listed on long lists are regarded here as being not under consideration, while species which are sampled, in progress or on priority lists are regarded as under consideration. If you are uncertain, please contact Thomas Marcussen and ask for advice.

You can check this on GoaT if you do not know (tutorial).

Taxonomic representation on ToL* (required)

○ Genus ○ Family ○ Order ○ Class ○ Phylum

The Earth Biogenome Project (EBP) has as its first goal to sample and generate reference genomes for family representatives. Below choose the taxonomic level of relationship to closest relative with a sequenced reference genome. For example, if one or more reference genomes are known for species from the same genus, choose genus, if it is from the same family, choose family and so forth. Please use the NCBI taxonomy.

You can check this on GoaT if you do not know (tutorial).

Genome size* (required)

○ 500 Mb or less ○ 501 to 1000 Mb ○ 1001 to 1500 Mb ○ 1501 to 2000 Mb ○ 2001 to 2500 Mb ○ 2501 to 3000 Mb ○ 3001 to 4000 Mb ○ 4001 to 5000 Mb ○ 5001 to 6000 Mb ○ 6001 to 8000 Mb ○ 8001 to 10000 Mb ○ 10001 to 15000 Mb ○ 15001 to 20000 Mb ○ 20001 Mb or more

Please provide the (estimated) genome size of the suggested species. You can check this on GoaT. if you do not know (tutorial).

Please indicate (if available) the estimated genome size or possible size range:

[ ]

Availability of voucher specimen* (required)

○ Yes, I will provide a physical voucher and photo ○ Yes, I will provide a digital voucher (photo)
○ No, I will not provide a voucher

BGE will not sequence species that lack a voucher. Ideally, the voucher specimen should be the specimen from which the tissues for DNA, RNA and Hi-C extraction have been taken. These remnants should enable morphological identification. For small species/specimens that are completely destroyed/consumed in the extraction, a proxy voucher from the same population/clone and preferably from the same collection event, and if relevant of the same sex, should be provided. A photo of the original specimen should be provided in any case. This photo can also serve as a digital voucher if due to size or ethical reasons the specimen cannot be vouchered (e.g., for threatened or endangered taxa).

Do you intend to provide a voucher?

Availability of a sample for biobanking* (required)

○ Yes, it will be possible ○ No, it will not be possible

BGE aims at providing tissue for biobanking, but it is not an exclusion or prioritization criterion. Ideally, this tissue comes from the specimen from which samples for DNA, RNA and Hi-C extraction have been taken. For small species/specimens that are completely destroyed/consumed in the extraction, a specimen from the same population/clone and preferably from the same collection event, can be provided.

Will it be possible to provide such a tissue sample in addition to the samples needed for sequencing and barcoding?

Nativity status in Europe* (required)

○ Native and occurs in the wild ○ Introduced and occurs in the wild ○ Introduced and kept in captivity or cultivation

BGE aims to sequence species that occur in the wild in continental Europe including surrounding waters, either as native or introduced.

Within Europe and international waters my species is:

Procedure of species identification (multiple choices are possible)* (required)

☐ Four-eyes principle (more than one person identifies independently the species) ☐ Available relevant keys for the region of collection ☐ Taxonomic expert for the group ☐ Species identification by DNA barcode before the submission of the sample ☐ None of the above

As the goal of EBP is to sequence reference genomes for each eukaryotic species, the species need to be identified with certainty. Please select all the procedures of species identification, which apply to the identification of your suggested species.

Certainty of species identification

○ Known taxonomic problems ○ No taxonomic problems

The certainty of identification can also be challenging due to known "taxonomic problems", such as species complexes, cryptic species or a long-standing lack of taxonomic revision, and without the capacity to identify the species with certainty given the procedures in the previous point. If you are aware of such problems for the suggested species, please indicate this.

Type locality* (required)

○ Collection at or close to type locality ○ Collection not close to type locality ○ Type locality not known or not yet attributed

Specimens close to the type locality are taxonomically preferable as they are more likely to be similar to the holotype of the species and hence its name bearer. If a well-defined type locality is present for the species, please indicate if the specimens will or have been collected from the type locality or close to it.

Country of sample site* (required)

ERGA as a European-wide society and BGE as an EU consortium aim at generating reference genomes and increase competence in genomic methods for the whole of Europe. Therefore, from which country will the specimens of the species be collected?

For international waters, use the ISO code XZ.

Will the team providing this species and generating the genome for it include a researcher from the country where the species was collected?* (required)

Yes No

Genome team definition.

Leader of the genome team (Sample Coordinator)* (required)

Yes No

The goal of BGE/ERGA is also to ensure that the benefit of generating reference genomes and competence building is not only distributed across the European countries, but also across individual researchers. This is similar to the incentive the EU-Assemble and EU-Assemble+ programs had.

Has the supposed sample coordinator for this species (usually the sample provider) not yet led a genome team before that has gotten the sequencing of a species granted by BGE/ERGA resources (excluding hot spot sampling).

Sample Coordinator Gender* (required)

Prefer not to say Non binary/Trans Female Male

ERGA aims to follow the JEDI principles and increase the inclusiveness and diversity of the genome teams. We want to empower especially gender diversity in leading roles of genome teams. Please indicate the gender of the sample coordinator (if you wish to disclose it).

Researcher of underrepresented minority* (required)

Prefer not to say Identify as an underrepresented minority (aside from gender, e.g. refugees, Romani people) Do not identify as an underrepresented minority

Increasing diversity does not only apply to gender, but also to other underrepresented minorities in the society. Hence, please indicate if the sample coordinator is member of an underrepresented minority (if you wish to disclose it).

If you have chosen "Identify as an underrepresented minority", please provide the information which underrepresented minority it is

Diversity & inclusiveness of the genome team (Multiple choices are possible)* (required)

Genome team has support from non-scientific interests or organizations (e.g., NGO, indigenous council) Representative(s) of non-scientific interests or organizations are included in the genome team Indigenous people are included in the genome team Disabled/handicapped persons are

included in the genome team ☐ Citizen scientists are included in the genome team ☐ None of the above applies

Diversity and inclusiveness should not only be represented in the leadership position, but also in the genome team itself. Moreover, BGE/ERGA aims to broaden the impact of the generated genomes by including non-scientific stakeholders. Please indicate how diverse and inclusive your genome team is.

Application of the genomes* (required)

○ Less than a year ○ Less than two years ○ Less than three years ○ More than three years

The generation of reference genomes should not be an aim by itself, but also benefit the larger scientific and non-scientific community at large. The faster such an impact can be generated the better for the visibility of the goals and sensibility of the EBP approach in general. After it has become publicly available, how quickly will the genome be used in research beyond your genome team?

Community benefit* (required)

○ 0 ○ 1 ○ 2 ○ 3 ○ 4 ○ 5 or more

Besides a quick application of the reference genomes, it also is beneficial if the genomes are used by a variety of stakeholders and communities, increasing the reach and breadth of the application of genomic resources.

How many communities / stakeholders would benefit from the genome that are not part of the genome team?

Please name the different stakeholders and communities, so that we can get an overview of benefit of the genome for the society at large.

Sample availability* (required)

○ Collected ○ Collected and Extracted ○ Not Collected

Sample acquisition is a critical step in the process, and excessive delays will jeopardize delivery across the whole BGE-ERGA project. For all species, the project assumes that the collectors have ascertained and acquired any required legal permits (wildlife, protected area, CITES). Please indicate the collection status of your species.

Collected = Already collected and stored appropriately when the call is closed on October 1st (see below for comments on “appropriateness” of collection and storage methods) or in culture.

Collected and Extracted = HMW DNA and/or RNA already extracted and stored appropriately (see below).

Not Collected = If samples need to be collected, the Species abundance and access criteria below will be used to prioritize species. For species where samples have not been collected, seasonality must be indicated.

Species abundance* (required)

CWA RA CW R

If your species still needs to be collected, BGE-ERGA might prioritize species that are abundant and easily accessible.

CWA = Common, widespread and abundant

RA = Rare, but locally abundant

CW = Common, widespread but not abundant

R = Rare, difficult to find

Month delivery of material

October 31st 2023 November 30th 2023 December 31st 2023 January 31st 2024 February 29th 2024 March 31st 2024 April 30th 2024 May 31st 2024 June 30th 2024 July 31st 2024 August 31st 2024 September 30th 2024

Please indicate the date before the sample can or could be delivered to the sequencing centers.

Number of samples and sampling across tissues* (required)

A single specimen, with tissues separated into multiple samples One specimen for long read and long-range data, with secondary specimens for transcriptomics Multiple specimens with each sufficient for only one data modality

BGE/ERGA reference genome sequencing generally proceeds from samples from a single specimen. Since some species are small, generating all three kinds of data needed for a reference genome (long read, long range, and transcriptome) can be difficult. In the case of small species, it is acceptable to sequence transcriptomes from secondary specimens. It is also possible, but less optimal, to sequence Hi-C long-range data from secondary specimens (even pooling several specimens could be an option at this step). BGE/ERGA will prioritize species for which multiple specimens are available, with perhaps multiple samples per specimen, and where samples of distinct tissues are available. Our sampling code of practice recommends that as few specimens of each species as are needed are sampled, but within this aim we do recommend sampling >1 specimen in 8-10 samples. We emphasize especially ***sampling the heterogametic sex*** *where known.*

Select the applicable category.

Size of sample

VS S M L CELLS

BGE-ERGA intends to assemble genomes from single individuals, and thus requires sufficient extractable DNA from a single specimen for at least long read data generation. Please indicate how large the ***specimen*** *is (keep in mind that there can be multiple samples for each specimen). Please use this scheme for assessing the size*

- *“VS” for very small (< 2 mm)*
- *“S” for small (~red lentil sized, (3-4mm))*
- *“M” for medium (~yellow lentil/ladybird sized/5mm)*

- *"L" for large (>5mm, chickpea/bean sized)*
- *If the specimen is cells (single cell organisms or cell cultures), use "CELLS" and indicate the number of cells possible to provide*

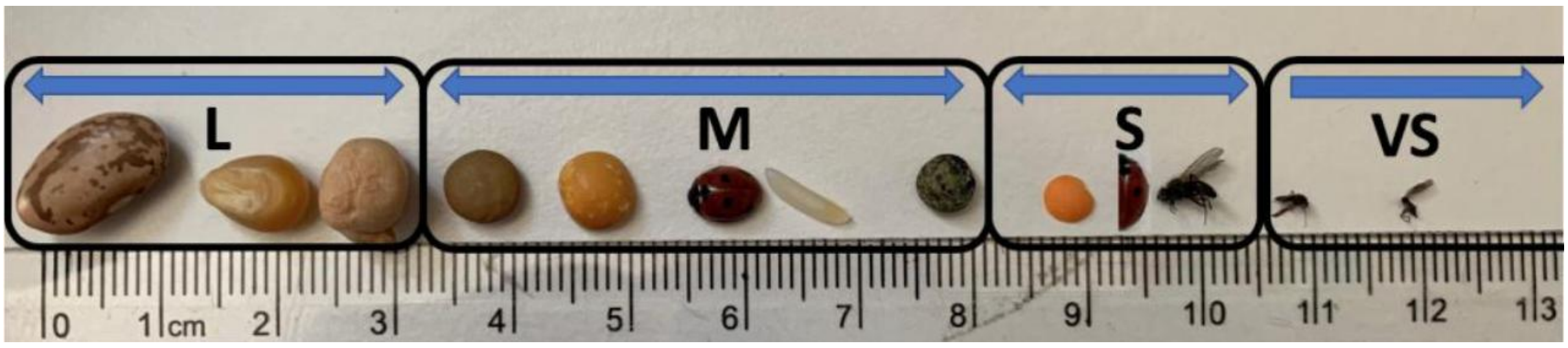

Method of preservation* (required)

○ Snap frozen (most appropriate) ○ Dry ice (most appropriate) ○ Ethanol/dry ice slurry ○ Lyophilised ○ Air dried ○ Qiagen allprotect ○ RNALater ○ RLT buffer ○ DMSO ○ DESS ○ Other

Good genomes cannot be generated from poor specimens, and BGE-ERGA thus prioritizes specimens that have been collected and preserved in a manner that assures excellent maintenance of DNA and RNA (and nuclear) integrity. Please specify how the living sample was preserved.

If a liquid solution such as QIAGEN Allprotect, RNALater, RLT Buffer, DMSO, or DESS etc. will/ has been used please specify the volume and concentration:

Sample preserved suitable for Hi-C* (required)

○ Yes ○ No

Generation of Hi-C long range data requires preservation of intact nuclei and thus samples for Hi-C must use compatible storage methods. The specimen should be snap frozen and maintained at <-70°C throughout.

Have the samples been snap frozen?

Sample preserved suitable for transcriptome sequencing

○ Snap frozen (most appropriate) ○ Dry ice (most appropriate) ○ Ethanol/dry ice slurry ○ QIAGEN Allprotect ○ RNALater ○ Other

Samples must be preserved in a way suitable for transcriptomics (Illumina RNAseq or Iso-seq).

Have the samples been preserved in one of the following ways?

Time elapsed from death to preservation

○ <5 mins ○ 5–30 mins ○ 31–60 mins ○ 1–2 hours ○ 2–8 hours ○ >8 hours

Some organisms may be held alive in captivity/culture for a period of time. This is not what is meant here, but rather the time that has passed since the sacrifice of the specimen to final preservation. Please specify the time (if you still need to collect the samples, provide an informed estimate here).

Storage of sample* (required)

○ Below -70 °C (e.g., dry ice shipper, -80 °C freezer) ○ Below -20 °C (e.g., regular freezer) ○ Below 4 °C (e.g., refrigerator) ○ None of the above

The storage of samples between collection and extraction is essential for high quality DNA, RNA and nuclear work. BGE-ERGA will prioritize samples that have a validated cold chain of storage since collection.

Please indicate the temperature of the validated cold chain.
